# Isomerization Reactions in Anionic Mesoionic Carbene‐Borates and Control of Properties and Reactivities in the Resulting Co^II^ Complexes through Agostic Interactions

**DOI:** 10.1002/anie.202013376

**Published:** 2020-11-17

**Authors:** Jessica Stubbe, Nicolás I. Neuman, Ross McLellan, Michael G. Sommer, Maite Nößler, Julia Beerhues, Robert E. Mulvey, Biprajit Sarkar

**Affiliations:** ^1^ Institut für Chemie und Biochemie Anorganische Chemie Freie Universität Berlin Fabeckstrasse 34–36 14195 Berlin Germany; ^2^ Instituto de Desarrollo Tecnológico para la Industria Química CCT Santa Fe CONICET-UNL Colectora Ruta Nacional 168, Km 472, Paraje El Pozo 3000 Santa Fe Argentina; ^3^ WestCHEM Department of Pure & Applied Chemistry University of Strathclyde Glasgow G1 1XL UK; ^4^ Lehrstuhl für Anorganische Koordinationschemie Institut für Anorganische Chemie Universität Stuttgart Pfaffenwaldring 55 70569 Stuttgart Germany

**Keywords:** cobalt, ligand rearrangement, mesoionic carbene, reactivity, spin state

## Abstract

We present herein anionic borate‐based bi‐mesoionic carbene compounds of the 1,2,3‐triazol‐4‐ylidene type that undergo C−N isomerization reactions. The isomerized compounds are excellent ligands for Co^II^ centers. Strong agostic interactions with the “C−H”‐groups of the cyclohexyl substituents result in an unusual low‐spin square planar Co^II^ complex, which is unreactive towards external substrates. Such agostic interactions are absent in the complex with phenyl substituents on the borate backbone. This complex displays a high‐spin tetrahedral Co^II^ center, which is reactive towards external substrates including dioxygen. To the best of our knowledge, this is also the first investigation of agostic interactions through single‐crystal EPR spectroscopy. We conclusively show here that the structure and properties of these Co^II^ complexes can be strongly influenced through interactions in the secondary coordination sphere. Additionally, we unravel a unique ligand rearrangement for these classes of anionic mesoionic carbene‐based ligands.

## Graphical abstract

C−N isomerization reactions on borate‐based anionic bi‐mesoionic carbene ligands are reported. The Co^II^ complexes with these ligands display geometries, spin states, and reactivities that are controlled by agostic interactions in the secondary coordination sphere.

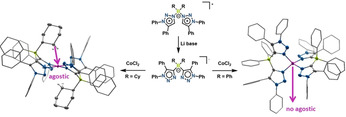

## Introduction

**Figure 1:**
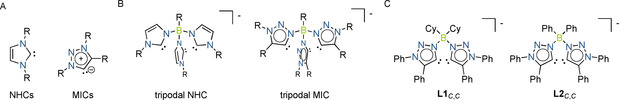
Structural examples of A: NHCs and MICs. B: borate‐based anionic NHCs and MICs. C: Bidentate borate‐based MICs of the form **L**_*C,C*_.

Borate‐based anionic N‐heterocyclic carbenes (NHCs) are well‐established as an important class of ligand.[^1^, ^2^, ^3^, ^4^, ^5^, ^6^, ^7^, ^8^, ^9^, ^10^, ^11^, ^12^, ^13^, ^14^, ^15^, ^16^, ^17^, ^18^, ^19^, ^20^, ^21^, ^22^] Such ligands have proven to be unique in terms of their ability to support small‐molecule activation at transition‐metal centers, as well as for their propensity to stabilize unusual metal oxidation states.[^23^, ^24^, ^25^, ^26^, ^27^, ^28^, ^29^, ^30^, ^31^, ^32^, ^33^, ^34^, ^35^, ^36^] 1,2,3‐Triazol‐5‐ylidene‐based mesoionic carbenes (MICs) are a relatively new addition to the larger class of NHCs (Figure 1 A).[^37^, ^38^, ^39^, ^40^, ^41^, ^42^]

**Scheme 1:**
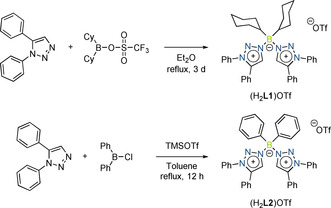
Synthetic approach for top: (H_2_ **L1**)OTf and bottom: (H_2_ **L2**)OTf.

In the past decade, metal complexes of MICs were shown to display excellent properties, e.g. in homogeneous catalysis,[^37^, ^38^, ^39^, ^40^, ^41^, ^42^, ^43^] in redox switchable catalysis,[^44^, ^45^, ^46^] in magnetism,^[47]^ and in photochemistry.[^48^, ^49^, ^50^, ^51^, ^52^, ^53^, ^54^, ^55^, ^56^, ^57^] Despite this progress in the development of MICs, the field is almost exclusively dominated by neutral MICs, even though there have been some recent examples of cationic MIC ligands.[^44^, ^45^, ^46^] To the best of our knowledge, there is just one report on a borate‐based anionic MIC compound of the triazolylidene‐type (Figure 1 B),^[58]^ which was synthesized from the corresponding 1,5‐regioisomer of the triazole.[^58^, ^59^] Given the unique electronic properties of MICs, and the well‐established utility of borate‐based NHCs, we were interested in developing bidentate borate‐based MICs of the form **L**_*C,C*_ (Figure 1 C). In the following, we present the synthesis of the bis‐triazolium salts (H_2_ **L1**)OTf and (H_2_ **L2**)OTf (Scheme 1), their deprotonation chemistry leading to C−N isomerization products, and the Co^II^ complexes of the isomerized ligands. Additionally, we show that the newly developed ligands can undergo unique backbone‐dependent strong agostic interactions with the Co^II^ center. Such interactions in the secondary coordination sphere of the metal center dictate its geometry, its spin state, and its chemical reactivity. A combined synthetic, crystallographic, spectroscopic (including single‐crystal EPR spectroscopy), and theoretical (wave‐function‐based methods) approach is used to investigate the compounds and their reactivity.

## Results and Discussions

**Scheme 2:**
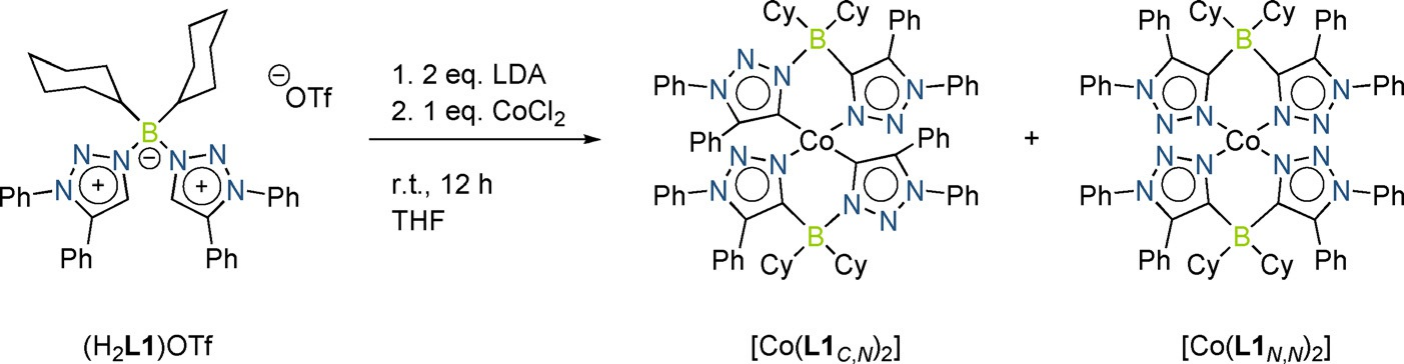
Synthetic approach for the complexes [Co(**L1**_*C*,*N*_)_2_] and [Co(**L1**_*N*,*N*_)_2_].

The triazolium‐substituted borate salt (H_2_ **L1**)OTf was synthesized in good yield by reacting 1,5‐diphenyl‐1*H*‐1,2,3‐triazole with dicyclohexyl‐trifluoromethanesulfonate‐borane (Scheme 1). With the aim of generating a bi‐MIC ligand from (H_2_ **L1**)OTf, we reacted this borate salt with CoCl_2_ in the presence of LDA at room temperature for 12 hours (Scheme 2). To our surprise, this reaction delivered a mixture of the complexes [Co(**L1**_*N*,*N*_)_2_] and [Co(**L1**_*C*,*N*_)_2_], which unfortunately could not be separated. The identity of these complexes was established by single‐crystal X‐ray diffraction studies (see below).

**Figure 2:**
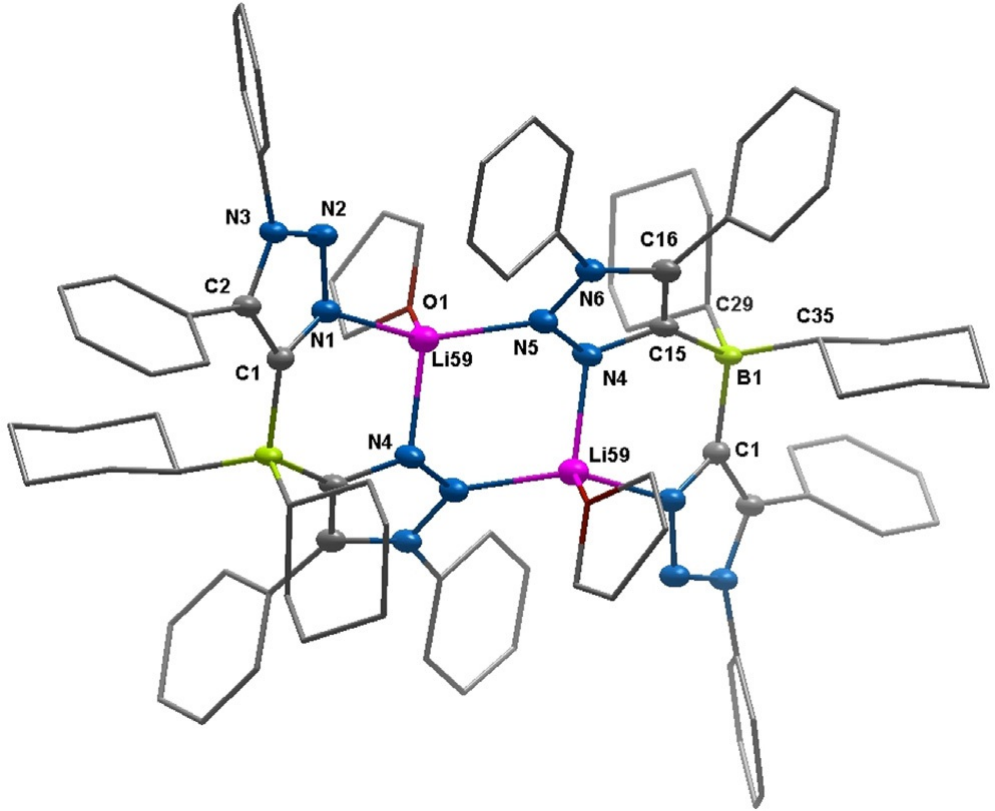
ORTEP representation of [Li(**L1**_*N*,*N*_)]_2_:^[74]^ ellipsoids drawn at 50 % probability. Solvent molecules and H‐atoms omitted for clarity. (For selected bond lengths and angles see supporting information Table S9)

**Scheme 3:**
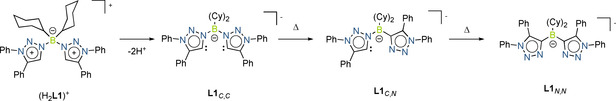
Deprotonation and isomerization of (H_2_ **L1**)OTf.

In these complexes either a single or a double C−N isomerization reaction took place on the ligand backbone during the deprotonation and the complexation process.[^60^, ^61^, ^62^, ^63^] With these observations in hand, we set out to decipher the parameters on which the C−N isomerization reaction is dependent (base, Co salt, temperature and so on). To this end (H_2_ **L1**)OTf was reacted with LiTMP (TMP=2,2,6,6‐tetramethylpiperidide) at −50 °C, and the mixture was slowly warmed up to room temperature over a period of 2 hours. A crystalline solid was obtained after purification in good yield. A ^1^H NMR spectrum of this compound did not display any signals corresponding to the C−H protons of the triazolium rings, indicating that the deprotonation was successful. The molecular structure in the crystal obtained through single‐crystal X‐ray diffraction studies shows the formation of a dilithium complex (Figure 2) and clearly depicts the formation of the C−N isomerization product (Scheme 3).

The bond lengths within the ligands are all in the expected range. The lithium centers are chelated through the nitrogen donors of the **L1**_*N*,*N*_ ligand. Additionally, there is an intermolecular coordination to an additional N‐atom (Li59−N5) from the second **L1**_*N*,*N*_ ligand. A THF molecule is also coordinated to the lithium centers resulting in a pseudo tetrahedral geometry at the metal ion. The two lithium ions together with the nitrogen donors form a six‐membered ring. The core of this structure has a fused seven‐ring 5:6:5:6:5:6:5 system.

**Figure 3:**
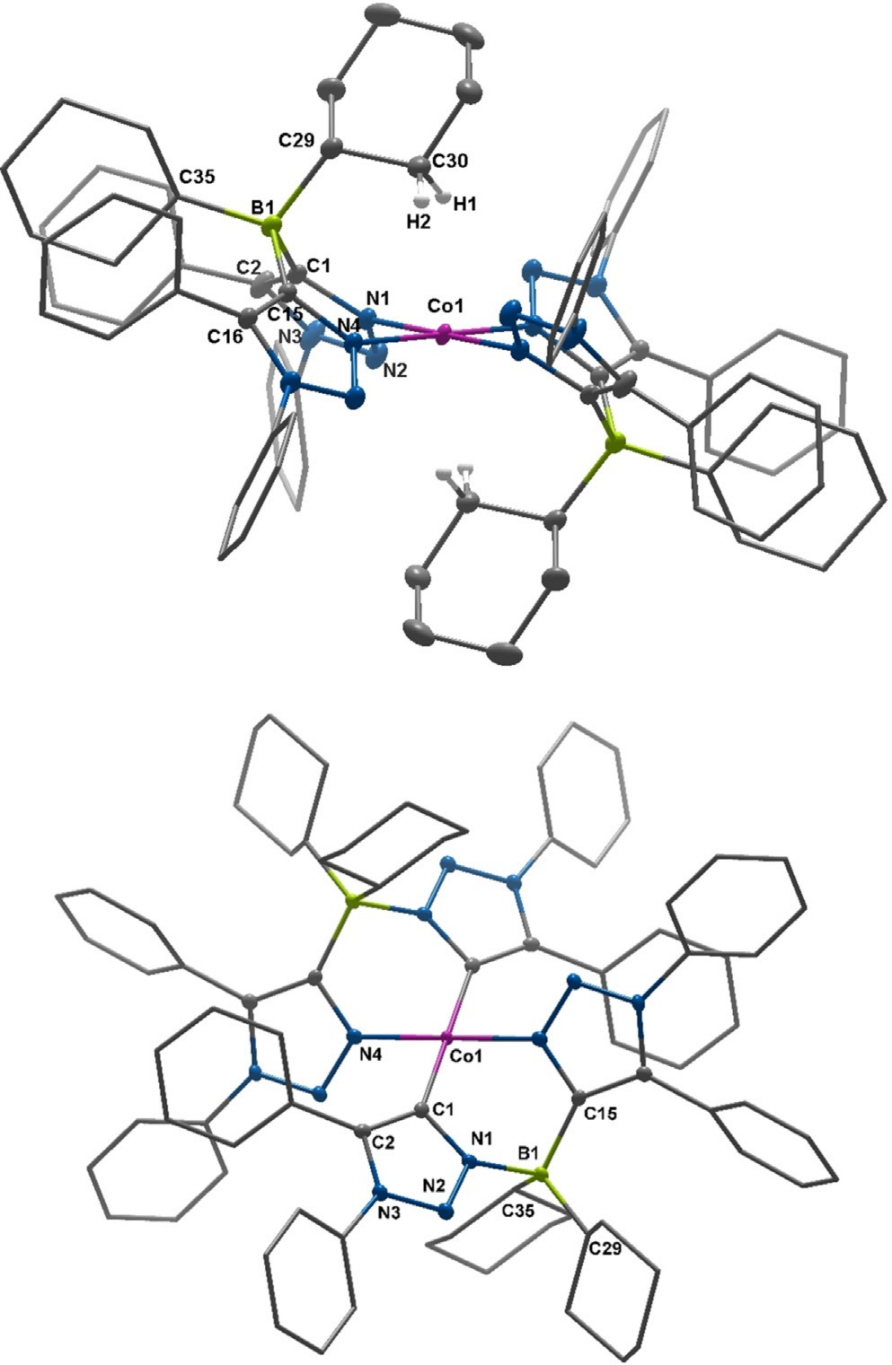
ORTEP representation of [Co(**L1**_*N*,*N*_)_2_] (top) and [Co(**L1**_*C*,*N*_)_2_] (bottom):^[74]^ ellipsoids drawn at 50 % probability. Solvent molecules and H‐atoms omitted (except agostic‐interacting CH_2_ groups) for clarity. (For selected bond lengths and angles see supporting information Tables S5 and S6)

These results clearly show that the presence of a cobalt salt (see above) is not necessary for the isomerization reaction at the ligand backbone. We next carried out a temperature‐dependent monitoring of the deprotonation and the isomerization reaction through multinuclear NMR spectroscopy. These results showed that with LiTMP, even at low temperatures, the deprotonation is almost instantaneous. Two different species are formed at low temperatures, which convert to one predominant species at room temperature, the signals of which display a good match with the signals of the isolated crystals (Figure S2). With the information obtained from these deprotonation studies, we set out to optimize the synthesis of the cobalt complex. (H_2_ **L1**)OTf was now reacted with LDA at 50 °C for 4 hours, cooled to room temperature, and the resulting mixture was stirred with CoCl_2_ for 12 hours. Work up of this mixture led to the isolation of only one type of complex, [Co(**L1**_*N*,*N*_)_2_], as crystalline material. The molecular structure in the crystal of [Co(**L1**_*N*,*N*_)_2_] shows that the cobalt center is coordinated by two nitrogen donors each from the two **L1**_*N*,*N*_ ligands (Figure 3, top).

The C−C, C−N and B−C bond lengths within the **L1**_*N*,*N*_ ligands are all within the expected range. Interestingly, the Co1−N1 and the Co1−N4 bond lengths of 1.907(1) and 1.918(1) Å are short and in a range expected for a low‐spin cobalt(II) ion. Additionally, the angles around the cobalt center are 86.6(1) and 93.4(1)°, which is very close to angles for a perfect square planar coordination geometry. The Co^II^ ion lies perfectly on the plane defined by the four N‐donor atoms. All these data point to a square planar, low‐spin Co^II^ center, which is extremely rare for Co^II^ complexes with non‐macrocyclic ligands. The complex [Co(**L1**_*N*,*N*_)_2_] displays several irreversible oxidation steps as revealed by cyclic‐voltammetry experiments. Additionally, two irreversible reduction steps are observed as well, the first of which becomes more reversible at higher scan rates (Figure S14). Inspired by the similarity in the geometry of the first coordination sphere of [Co(**L1**_*N*,*N*_)_2_] to square planar Co^II^–porphyrin complexes, we set out to investigate the reactivity of this complex with pyridine and dioxygen. To our surprise and disappointment, the complex [Co(**L1**_*N*,*N*_)_2_] did not react with either of the external substrates. As the single crystals of [Co(**L1**_*N*,*N*_)_2_] were relatively large (0.29×0.21×0.11 mm^3^) and of a well‐defined shape, we decided to perform single‐crystal EPR measurements on that complex.

**Figure 4:**
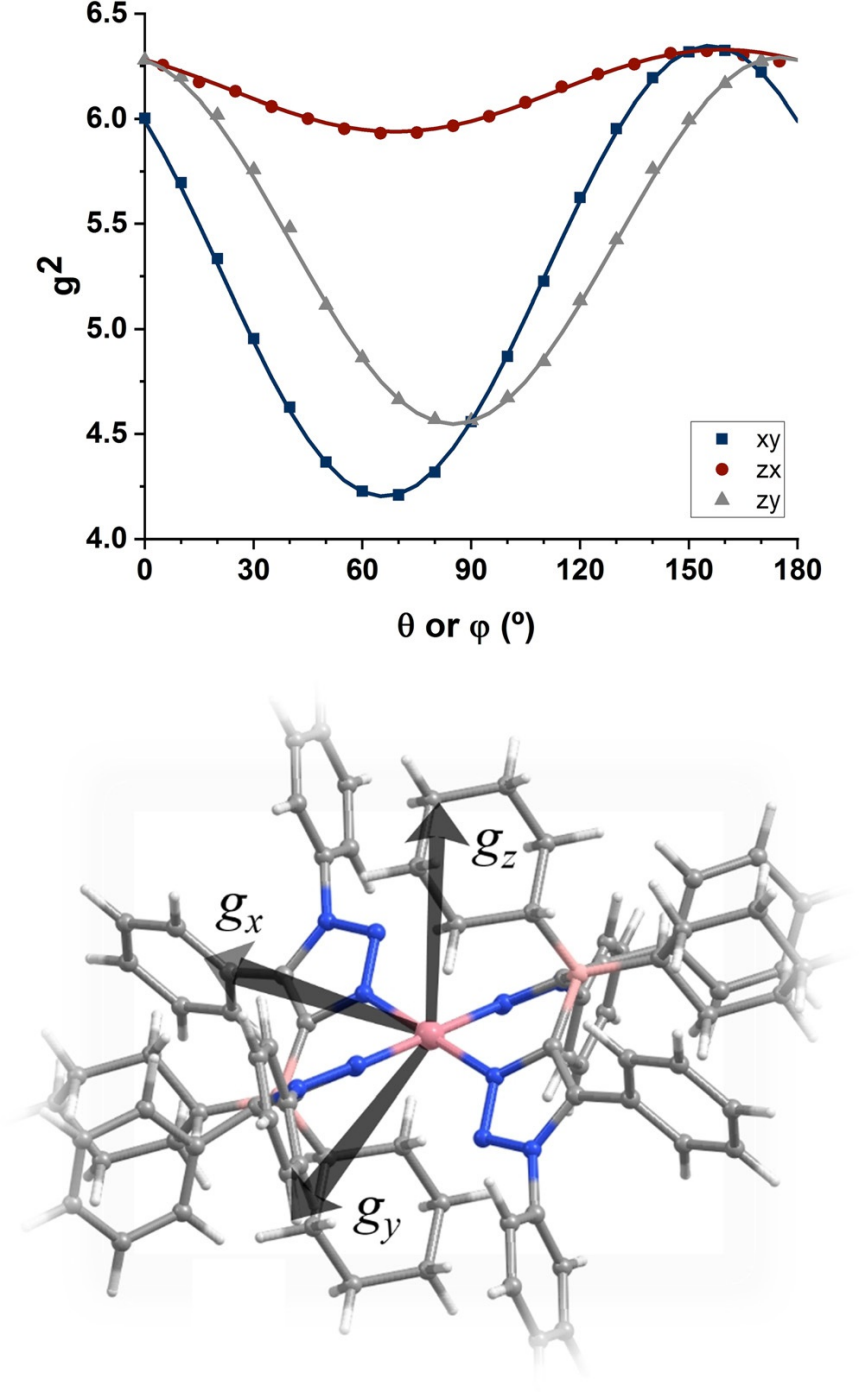
Angular variation of the crystal *g* ^2^‐value in three orthogonal planes in the experimental *xyz* coordinate system, and orientation of the principal axes of the **g**‐matrix in the molecular coordinate frame.

To achieve this, a single crystal was glued to a KCl cubic crystal, thus defining an experimental *xyz* coordinate system. The KCl cube was placed on a flat surface on a special quartz rod, which was introduced in the EPR spectrometer cavity, attached to a home‐made goniometer. Placing the KCl cube on each of three orthogonal faces and rotating the quartz rod allowed as to acquire EPR spectra for multiple crystal orientations in three orthogonal planes. Further details are given in the SI and in selected references.^[64]^ The spectra showed one set of eight well‐resolved hyperfine lines (*I*(^59^Co)=7/2) arising from a single cobalt(II) centre, which reveals the triclinic *P* $\bar{1}$ space group of the crystal. Selected single‐crystal spectra are shown in Figure S15 and the full set of spectra in the three experimental crystal planes is shown in Figure S16. The well‐resolved hyperfine splittings and low linewidths indicate that intermolecular magnetic dipolar and exchange interactions are negligible,[^65^, ^66^] which is congruent with the long Co−Co distances (12.702(1) Å) and with the presence of bulky cyclohexyl and phenyl groups which interact only weakly in the crystal. From the central position of the resonances we calculated the *g*‐value for each orientation, and from the average separation *a* (mT) between adjacent resonances, the *K*‐factor (*K*=*agμ*_B_/*h*, were *μ*_B_ is the Bohr magneton and *h* is Planck's constant) was calculated. Analysis of the angular dependence of the *g* ^2^ value and of the *g* ^2^ *K* ^2^‐value allowed us to obtain the **g**‐matrix and hyperfine **A**‐matrix, following procedures described elsewhere.[^64^, ^67^] The angular dependence of the *g* ^2^‐value and the molecular orientation of the **g**‐matrix are shown in Figure 4.

**Figure 5:**
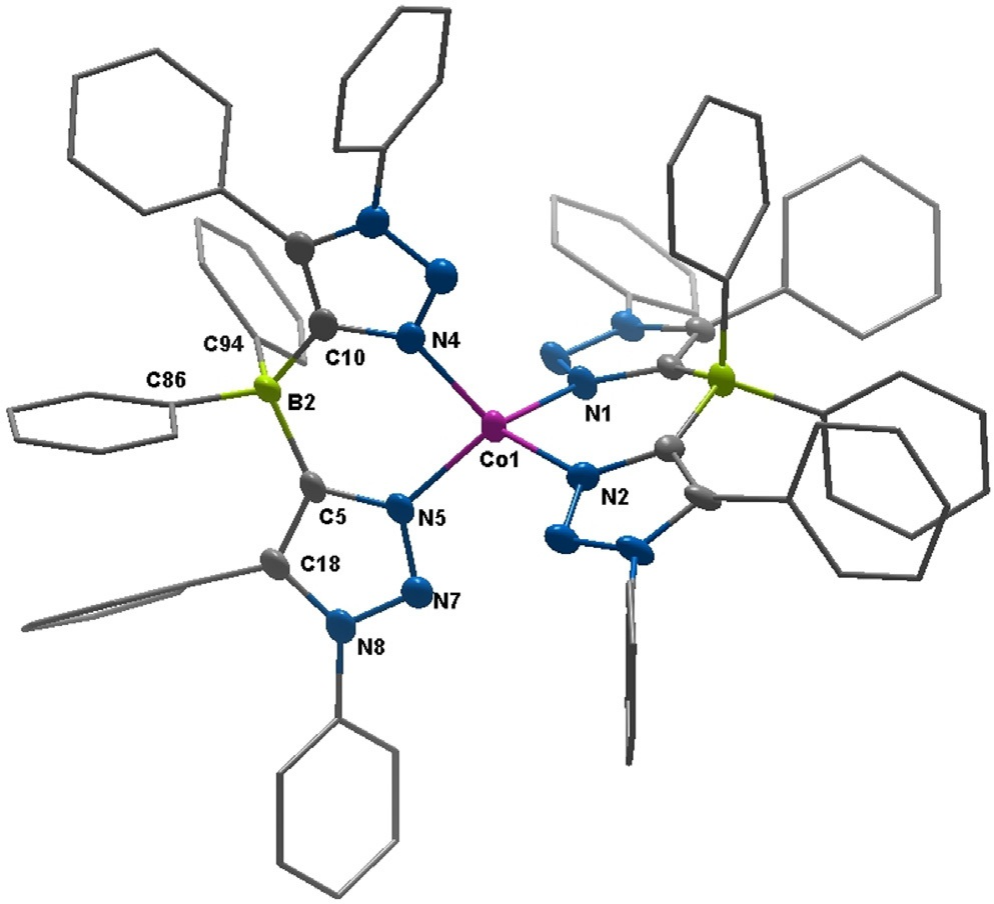
ORTEP representation of [Co(**L2**_*N*,*N*_)_2_]:^[74]^ ellipsoids drawn at 50 % probability. Solvent molecules and H‐atoms omitted for clarity. (For selected bond lengths and angles see supporting information Table S7)

To our surprise, the *g*‐values obtained from these measurements (2.045, 2.502, and 2.526) (see Table S1) did not match well with typical *g*‐values reported for square planar Co^II^–porphyrin complexes (for instance Co(*p*‐OCH_3_)TPP with *g*_∥_=1.79 and *g*_**⊥**_=3.29), but with *g*‐values for penta‐ or hexa‐coordinated Co^II^–porphyrin complexes containing additional axial ligands.^[68]^ We then went back and had a careful look at the molecular structure of [Co(**L1**_*N*,*N*_)_2_] in the crystal. We discovered that one “CH_2_‐group” each from the cyclohexyl substituents at the borate backbone of the **L1**_*N*,*N*_ ligands is oriented exactly at the axial coordination sites of the Co^II^ center (Figure 4, top). The Co−H distances are 2.31(3) and 2.69(2) Å, pointing to strong agostic interactions between the Co^II^ center and the substituents on the secondary coordination sphere of the ligand backbone.^[69]^ The consideration of these interactions was also important to get a good agreement between the experimental and the calculated EPR parameters (see theory section below). To the best of our knowledge, this is the first instance in which single crystal EPR spectroscopy has been used to unravel agostic interactions in a metal complex. The complex with [Co(**L1**_*C*,*N*_)_2_] (Figure 3, bottom), where only one C−N isomerization has taken place on the ligand backbone, was also obtained as single crystals. The coordination geometry around the Co^II^ center in that complex as well as other structural parameters are very similar to [Co(**L1**_*N*,*N*_)_2_] (Figure 3, bottom and Table S6). However, we were not able to obtain [Co(**L1**_*C*,*N*_)_2_] in the pure form as bulk material and hence this complex will not be discussed any further herein. The detailed analysis of the molecular structure in the crystal, the EPR parameters, and the reactivity studies (basically the lack of reactivity) of [Co(**L1**_*N*,*N*_)_2_] led us to hypothesize that the geometry, the spin state, and the reactivity of this metal complex are controlled by the strong non‐covalent (agostic) interactions in the secondary coordination sphere. Additionally, in the IR spectrum of the complex [Co(**L1**_*N*,*N*_)_2_] a weak and broad signal is observed at 2620 cm^−1^, which is also an indication of the presence of an agostic interaction in this complex (Figure S12 and S13). Such a signal is absent from all the other compounds investigated here, lending credence to the assignment of this band to the presence of agostic interactions. Calculations reproduce this band with reasonable accuracy (Figure S24 and discussion in the SI). In order to validate this hypothesis of the effect of agostic interactions on the properties of the cobalt complex, we decided to synthesize the ligand (H_2_ **L2**)OTf, which has two phenyl substituents (instead of cyclohexyl) on the borate backbone of the ligand.

(H_2_ **L2**)OTf was synthesized by the reaction of 1,5‐diphenyl‐1*H*‐1,2,3‐triazole with diphenylchloro‐borane, followed by the addition of trimethylsilyl triflate, in good yield (Scheme 1, bottom). The deprotonation of (H_2_ **L2**)OTf with LDA and further reaction with CoCl_2_ delivered [Co(**L2**_*N*,*N*_)_2_] in reasonable yield. Whereas the crystals of [Co(**L1**_*N*,*N*_)_2_] are yellow in color, [Co(**L2**_*N*,*N*_)_2_] is pink in the crystalline state. A look at the molecular structure of [Co(**L2**_*N*,*N*_)_2_] in the crystal shows that the Co^II^ center is coordinated by two N‐atoms each from both the ligands (Figure 5).

Thus, as expected, two C−N isomerization reactions have taken place on the backbone of each of the ligands. All four Co−N bond lengths in [Co(**L2**_*N*,*N*_)_2_] are around 1.973(6) Å, and are thus substantially longer than the Co−N bond lengths in [Co(**L1**_*N*,*N*_)_2_] (1.913(3) Å). Additionally, the N‐Co‐N bond angles around the metal center are 94.1(1), 115.9(1), 116.7(1), and 94.7(1)°. These values indicate a distorted tetrahedral geometry around the Co^II^ center with the distortion being imposed by the chelating nature of the **L2**_*N*,*N*_ ligand. Additionally, no agostic or other non‐covalent interactions are observed in the secondary coordination sphere of the cobalt center in this complex. All the above data point towards a high‐spin tetrahedral Co^II^ center in [Co(**L2**_*N*,*N*_)_2_]. Accordingly, no EPR signals were observed for this compound even at liquid nitrogen temperatures. Thus, it is seen that the phenyl substituents on the borate backbone of **L2**_*N*,*N*_ do not display any agostic interactions with the Co^II^ center. This result is strikingly different from what was shown above for the complex [Co(**L1**_*N*,*N*_)_2_]. The agostic interactions on the secondary coordination sphere thus control the geometry (square planar vs. tetrahedral), the spin state (low spin vs. high spin), and the EPR properties (presence or absence of an EPR signal at liquid nitrogen temperatures) of the complexes [Co(**L1**_*N*,*N*_)_2_] and [Co(**L2**_*N*,*N*_)_2_].

**Figure 6:**
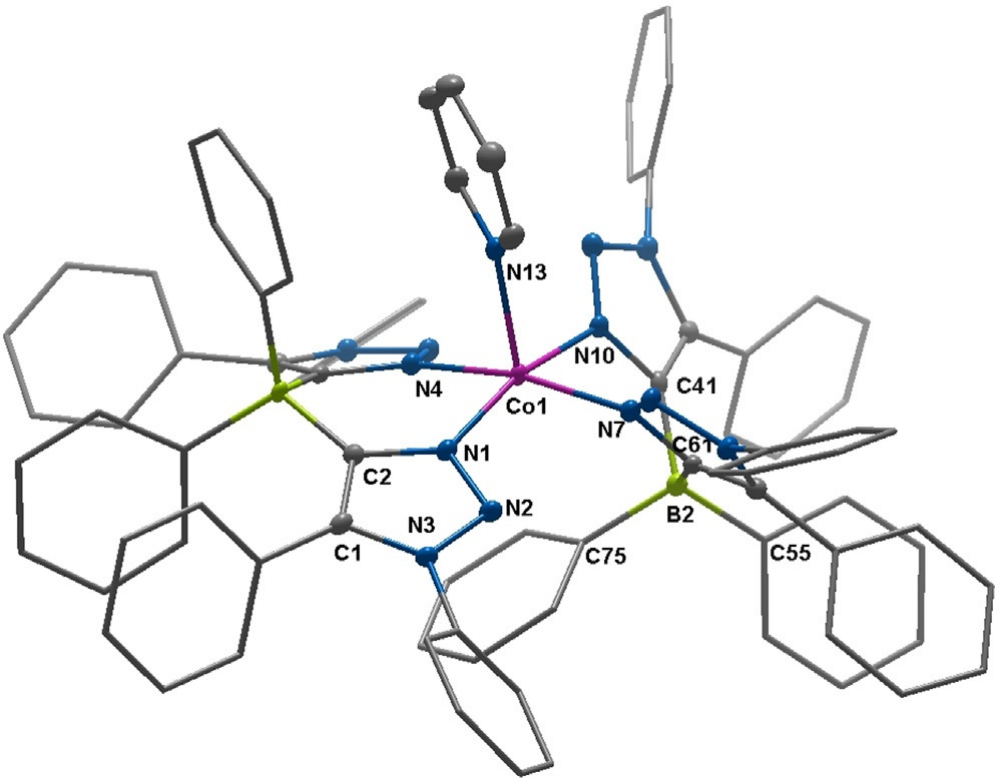
ORTEP representation of [Co(**L2**_*N*,*N*_)_2_py]:^[74]^ ellipsoids drawn at 50 % probability. Solvent molecules and H‐atoms omitted for clarity. (For selected bond lengths and angles see supporting information Table S8)

As was mentioned above, [Co(**L1**_*N*,*N*_)_2_] does not show any reactivity towards the tested external substrates despite possessing a formal square planar coordination geometry at the Co^II^ center, and a low‐spin state. We attributed the absence of this reactivity to the blocking of the axial coordination sites through agostic interactions. Gratifyingly, complex [Co(**L2**_*N*,*N*_)_2_], which does not display any agostic interactions, reacts spontaneously with pyridine (as seen in control EPR experiments, see SI, Figure S20). A proper reaction of [Co(**L2**_*N*,*N*_)_2_] with pyridine led to the quantitative isolation of the complex [Co(**L2**_*N*,*N*_)_2_py]. The Co^II^ center in [Co(**L2**_*N*,*N*_)_2_py] has a distorted square pyramidal geometry (Figure 6) with a *τ* value of 0.44.

**Figure 7:**
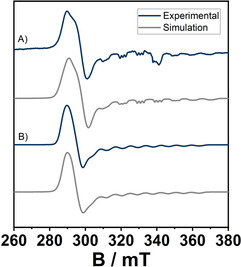
EPR spectra of A): [Co(**L2**_*N*,*N*_)_2_] in DCM with an excess of **pyridine** at 100 K. and B): isolated [Co(**L2**_*N*,*N*_)_2_py] as a powder at 100 K.

The Co−N bond lengths in the equatorial plane are between 1.938(1) and 1.969(1) Å. The Co−N distance to the pyridine‐N atom is 2.138(1) Å. Complex [Co(**L2**_*N*,*N*_)_2_py] displays an EPR signal both in the solid state and in solution at 100 K (Figure 7).

**Figure 8:**
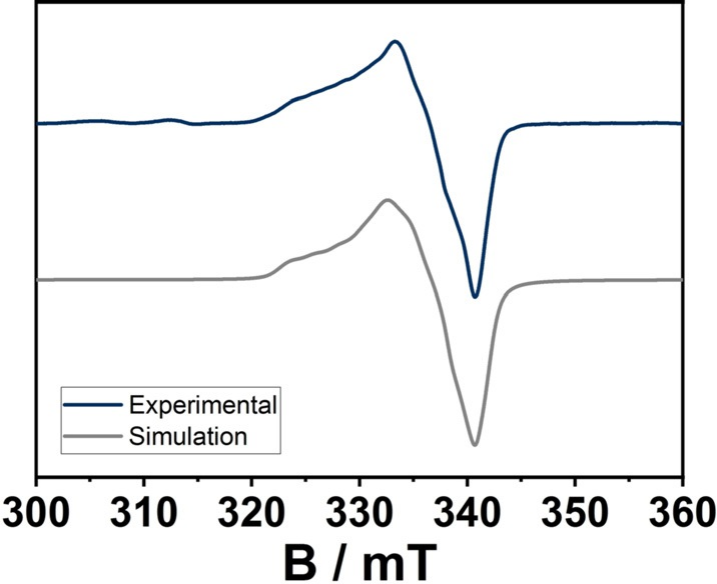
EPR spectra of [Co(**L2**_*N*,*N*_)_2_] in DCM in the presence of **O_2_** at 100 K.

The EPR spectrum obtained from a powder polycrystalline sample of [Co(**L2**_*N*,*N*_)_2_py] was simulated with *g*‐values of 2.298, 2.290, and 2.028 and *A*‐values (^59^Co, *I*=7/2) of 17.5, 19.2, and 243 MHz. The observed bond lengths, the near axial *g*‐anisotropy, and a large hyperfine coupling to the cobalt nucleus along the *z*‐direction are all signs of a low‐spin Co^II^ center in [Co(**L2**_*N*,*N*_)_2_py]. The EPR spectrum recorded in frozen solution has similar *g*‐ and *A*‐values. Additionally, for the spectrum in frozen solution, the hyperfine coupling to the axial pyridine‐N nucleus is resolved as well (Figure 7). Thus, on coordination of a pyridine ligand, the spin state of the Co^II^ ion changes from high spin in [Co(**L2**_*N*,*N*_)_2_] to low spin in [Co(**L2**_*N*,*N*_)_2_py]. Prompted by the reactivity of [Co(**L2**_*N*,*N*_)_2_] with pyridine, and its extremely air‐sensitive nature, we decided to react [Co(**L2**_*N*,*N*_)_2_] with O_2_. The new compound formed delivered an EPR signal which was simulated with *g*‐values of 1.993, 2.024, and 2.040 and *A*‐values (^59^Co, *I*=7/2) of 9, 13, and 65 MHz (Figure 8). These EPR parameters match very nicely with EPR parameters reported in the literature for a distorted square pyramidal Co^III^ center which is bound to an axial superoxide (O_2_^.−^) radical.[^70^, ^71^] Thus, the complex [Co(**L2**_*N*,*N*_)_2_] appears to be able to activate and bind O_2_.

**Figure 9:**
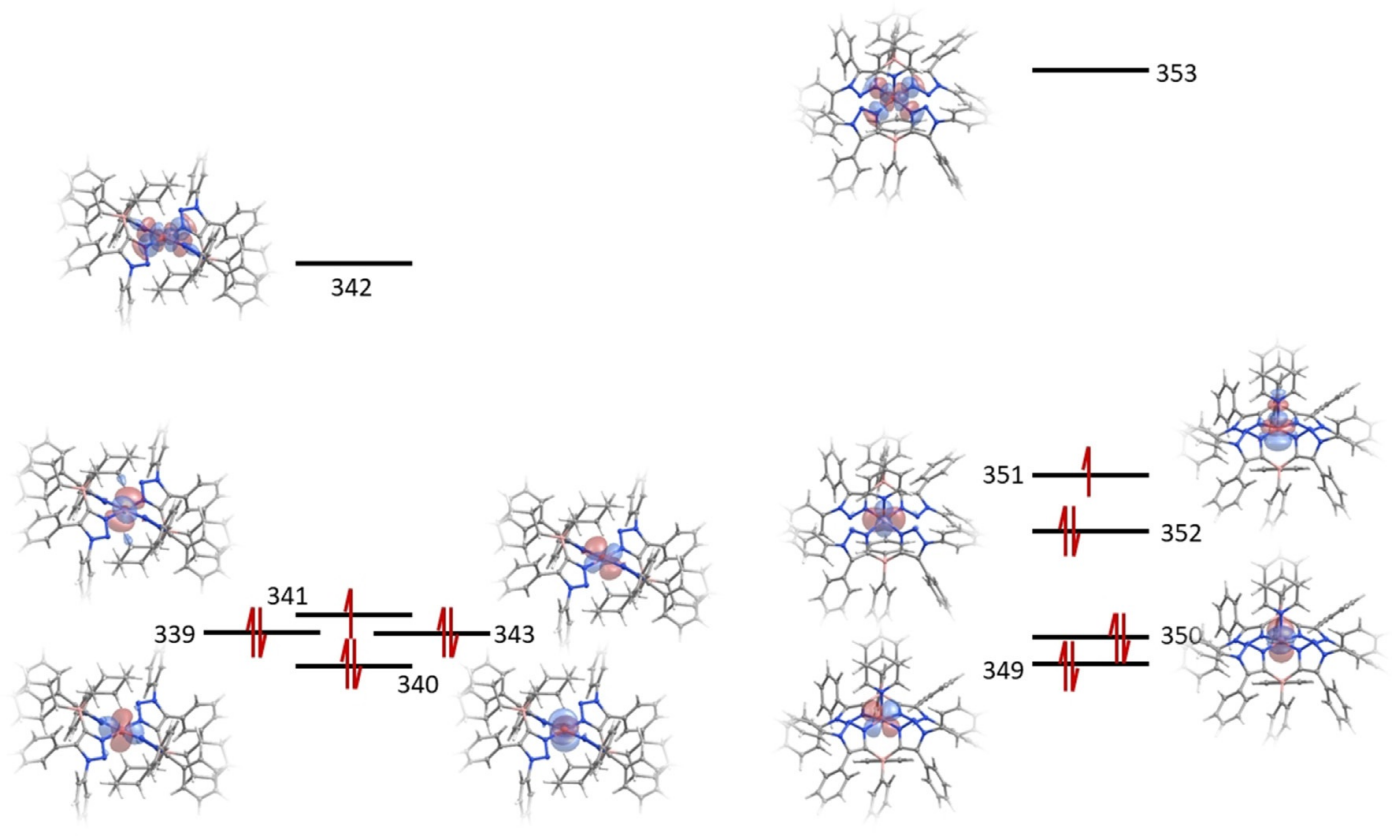
Orbital energy diagram for the 3d orbitals of [Co(**L1**_*N*,*N*_)_2_] and [Co(**L2**_*N*,*N*_)_2_py].

In order to correlate the results from EPR experiments with the electronic structure and ligand field energetics of the different cobalt complexes, including a truncated form of [Co(**L1**_*N*,*N*_)_2_]: [Co(**L1**_*N*,*N*_)_2_]^Tr^, where the apical CH_2_ groups directly above and below the Co^II^ atom were removed from the cyclohexyl substituents, we performed complete‐active space self‐consistent field (CASSCF) calculations, with multi‐reference perturbation theory (NEVPT2) to take into account dynamical correlation effects, using the ORCA program.^[72]^ We found that for complexes [Co(**L1**_*N*,*N*_)_2_] and [Co(**L2**_*N*,*N*_)_2_py] the low‐spin multiplicity configuration was the ground state. The CASSCF/NEVPT2 calculations show that the ground state configurations for [Co(**L1**_*N*,*N*_)_2_], [Co(**L1**_*N*,*N*_)_2_]^Tr^, and [Co(**L2**_*N*,*N*_)_2_py] contain the unpaired electron mainly in the d${}_{z{}^{2}}$ orbital, with the first two excited configurations corresponding to excitations from the d${}_{z{}^{2}}$ to d_*xz*_ and d_*yz*_ orbitals, respectively (Figure 9).

The energy difference between the ground d${}_{z{}^{2}}$ and the first two excited states determines the deviation of the *g_x_* and *g_y_* values from *g_e_=*2.^[73]^ The NEVPT2 energy difference for [Co(**L1**_*N*,*N*_)_2_] is in the 5400–5500 cm^−1^ range, while for the truncated form [Co(**L1**_*N*,*N*_)_2_]^Tr^, it is in the 2800–3050 cm^−1^ range and for [Co(**L2**_*N*,*N*_)_2_py] it is in the 9300–9560 cm^−1^ range. These calculations were able to reproduce the experimental *g*‐values with remarkable accuracy (Table 1), therefore providing strong evidence that the energy difference between the ground and first two excited configurations, and therefore the *g_x_*‐ and *g_y_*‐values, are heavily influenced by the strength of the apical ligand field.

**Table 1:** Experimental and calculated (CASSCF/NEVPT2) *g*‐values for [Co(**L1**_*N*,*N*_)_2_] and [Co(**L2**_*N*,*N*_)_2_py], as well as a truncated form of [Co(**L1**_*N*,*N*_)_2_] ([Co(**L1**_*N*,*N*_)_2_]^Tr^).

	[Co(**L1**_*N*,*N*_)_2_] Experimental (Single Crystal EPR)	**[Co(L1**_***N***,***N***_ **)_2_]** CASSCF/NEVPT2	[Co(**L1**_*N*,*N*_)_2_]^Tr^ CoN4 no apical CASSCF/NEVPT2	[Co(**L2**_*N*,*N*_)_2_py] CASSCF/NEVPT2	[Co(**L2**_*N*,*N*_)_2_py] Experimental (powder)
*g_x_*	**2.502**	2.512	2.815	2.296	**2.298**
*g_y_*	**2.526**	2.529	2.891	2.319	**2.290**
*g_z_*	**2.045**	2.004	1.965	1.994	**2.028**

The EPR results and CASSCF/NEVPT2 calculations thus indicate that the agostic interaction between the apical ‐CH_2_‐groups and the Co^II^ ion not only influence the coordination geometry and spin state, but also directly affect ligand field splittings and modify the *g*‐values.

## Conclusion

In conclusion, we have presented here two new borate‐based triazolium salts, which undergo unique C−N isomerization reactions at the ligand backbone on deprotonation. Co^II^ complexes with these isomerized ligands display unusual behavior. We could conclusively show that the geometry, the spin state, the spectroscopic properties, and the reactivity of these complexes can be controlled through unique agostic interactions between the substituent on the borate backbone and the cobalt center in its secondary coordination sphere. To the best of our knowledge, we have for the first time investigated the phenomenon of agostic interactions through single‐crystal EPR spectroscopy. The results presented here open up new avenues for these anionic borate‐based bi‐mesoionic carbene ligands for controlling spin state and reactivity at the metal center including small‐molecule activation. Such studies, including complexation with other transition metals, are currently ongoing in our laboratories.

## Conflict of interest

The authors declare no conflict of interest.

## Supporting information

As a service to our authors and readers, this journal provides supporting information supplied by the authors. Such materials are peer reviewed and may be re‐organized for online delivery, but are not copy‐edited or typeset. Technical support issues arising from supporting information (other than missing files) should be addressed to the authors.

Supplementary
