# Supplementary material for: Isomerization Reactions in Anionic Mesoionic Carbene‐Borates and Control of Properties and Reactivities in the Resulting CoII Complexes through Agostic Interactions
Source: Angew Chem Int Ed Engl. 2020 Nov 17;60(1):499–506. doi: 10.1002/anie.202013376 (PMC7839553; doi:10.1002/anie.202013376)
Supplement: Supplementary file 1 — Supplementary [file ANIE-60-499-s001.pdf]

Angewandte Chemie
Eine Zeitschrift der Gesellschaft Deutscher Chemiker

Supporting Information

Isomerization Reactions in Anionic Mesoionic Carbene-Borates and Control of Properties and Reactivities in the Resulting Co^{II} Complexes through Agostic Interactions

*Jessica Stubbe, Nicolás I. Neuman, Ross McLellan, Michael G. Sommer, Maite Nößler, Julia Beerhues, Robert E. Mulvey, and Biprajit Sarkar**

anie_202013376_sm_miscellaneous_information.pdf

Supporting Information

1. Synthetic procedures 2
    1.1. Ligand synthesis 4
    1.2. Deprotonation experiments 6
    1.3. Complex synthesis 9
2. NMR Spectra 12
3. IR Spectroscopy 16
4. Cyclic Voltammetry 17
5. EPR 18
6. Electronic Structure Calculations 24
7. Crystal Structures & Crystallographic Data 29
8. Molecular Geometries used in Electronic Structure Calculations 36
9. References 45

1. Synthetic procedures and instrumentation

Unless otherwise noted, all reactions were performed using standard Schlenk-line techniques under an inter atmosphere of argon (Linde, Argon 4.8, purity ≥ 99.998) or an MBraun glove box fitted with a gas purification and recirculation unit. Commercially available chemicals were used without further purification. THF, diethyl ether, toluene and *n*-hexane were dried and distilled from sodium, dichloromethane and acetonitrile from phosphorus pentoxide. Other solvents were available from MBRAUN MB-SPS-800 solvent system and additionally degassed using standard techniques.

^{1}H NMR and ^{11}B NMR were recorded on Joel ECS 400 or JOEL 400R spectrometer. Proton decoupled ^{13}C NMR were recorded on AVANCE700 proton resonance spectrometer.

NMR spectra based on rearrangement experiments were recorded on a Bruker AV3 or AV 400 MHz spectrometer operating at 400.13 MHz for ^{1}H; 100.62 MHz for ^{13}C; 155.47 MHz for ^{11}B; 376.46 MHz for ^{7}Li. All ^{13}C spectra were proton decoupled. ^{1}H and ^{13}C NMR spectra were referenced against the appropriate solvent signal. ^{7}Li NMR spectra were referenced against LiCl in D_2O at 0.00 ppm and ^{11}B spectra were referenced against $BF_3{\cdot}OEt_2$ in $CDCl_3$ at 0.00 ppm

Chemical shifts are reported in ppm (relative to the TMS signal) with reference to the residual solvent peaks.[1] Multiplets are reported as follows: singlet (s), duplet (d), triplet (t), quartet (q), quintet (quint), and combinations thereof. NMR experiments were conducted in J. Young's NMR tubes oven dried and flushed with argon prior to use.

Mass spectrometry was performed on an Agilent 6210 ESI-TOF.

Cyclic voltammograms were recorded with a PAR VersaStat 4 potentiostat (Ametek) by working in anhydrous and degassed tetrahydrofuran with 0.1 M NBu_4PF_6 (dried, > 99.0%, electrochemical grade, Fluka) as supporting electrolyte. Concentrations of the compounds were about $1{\cdot}10^{-4}$ M. A three-electrode setup was used with a glassy carbon or gold working electrode, a coiled platinum wire as counter electrode, and a coiled silver wire as a pseudo-reference electrode. The ferrocene/ferrocenium couple was used as internal reference.

FTIR-ATR spectra were recorded with a Thermo Scientific™ Nicolet™ iS™10 FT-IR spectrometer equipped with a smart orbit unit.

X-ray data were collected on a Bruker Smart AXS or Bruker D8 Venture systems at 140(2) K or 100(2) K, respectively, using graphite-monochromated $Mo_{K\alpha}$ radiation (λ_α= 0.71073 Å). The strategy for the data collection was evaluated by using the Smart software. The data were collected by the standard omega scan or omega + phi scan techniques, and were scaled and reduced using Saint+ and SADABS software. The structures were solved by direct methods using SHELXS-97 or intrinsic phasing using SHELXL-2014/7 and refined by full matrix least-squares, refining on F^2. Non-hydrogen atoms were refined anisotropically.[2-8] Crystallographic data of [Li(**L1*N,N***)]$_2$ were collected on an Oxford Diffraction instrument with $Cu_{K\alpha}$ radiation (λ = 1.54184 Å). Structures were solved using SHELXS-97[4] and the software OLEX2,[9] while refinement was carried out on F2 against all independent reflections by the

full matrix least-squares method using the SHELXL-97 program. All non-hydrogen atoms were refined using anisotropic thermal parameters.

Room temperature X-band CW-EPR spectra of an oriented single crystal [Co(**L1**$_{N,N}$)$_2$] were acquired on a Magnettech MS5000 EPR spectrometer equipped with a rectangular TE 102 cavity. A single crystal was adhered with vacuum grease on one of the faces of a cubic KCl crystal (approximately 2x2x2 mm), thus defining an orthogonal coordinate system *xyz*. The KCl crystal was placed on a flat surface inside a cavity performed on a 4 mm diameter quartz rod. The rod was inserted in the cavity of the EPR spectrometer, solidary to a home-made goniometer. This set up allowed us to acquire EPR spectra from 0º to 180º, in 5º intervals, on three orthogonal planes (*xy*, *zx*, *zy*), by subsequently placing down a different face of the KCl cube.

1.1.Ligand synthesis

Attention: Organic azides are potentially explosive and should be handled with great care and only in small quantities. Although we never experienced any problems, the reaction with organic azides should only be attempted in amounts up to 2 mmol!

[Cy_2B(diPhtz)$_2$](OTf): (H_2**L1)**OTf

Phenyl azide and 1,5-diphenyl-1*H*-1,2,3-triazole (diPhtz) were synthesized according to a literature procedure.[10]

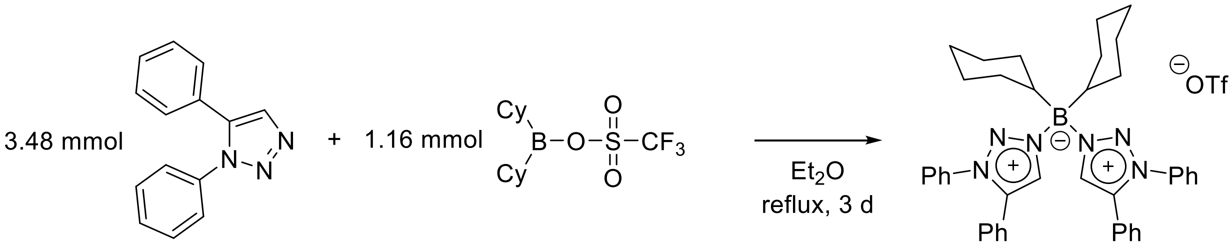

Under an inert atmosphere of argon 1,5-diphenyl-1*H*-1,2,3-triazole (770 mg, 3.48 mmol) and dicyclohexyl-trifluoromethanesulfonate-borane (378 mg, 1.16 mmol) were dissolved in dry diethyl ether (60 mL) and refluxed for 24 h under an inert atmosphere. During the reaction, the product precipitated as white solid. The white solids were collected by filtration, washed with diethyl ether and dried under high vacuum to give the desired product in good yields of 87%.

^{1}H NMR (400 MHz, $CDCl_3$, 20 °C) δ 8.59 (s, 2 H, trz-*H*), 7.63–7.32 (m, 10 H, phenyl-*H*), cyclohexyl-*H*: 1.91–1.60 (m, 10 H), 1.45–1.18 (m, 6 H), 1.11–0.92 (m, 2 H), 0.66–0.43 (m, 4 H) ppm.

^{13}C NMR (176.1 MHz, $CDCl_3$, 26.9 °C) δ 141.14 (quart-Ph*C*–N), 135.27 (trz-N–*C*–C), 131.47 (Trz-*C*–H), 131.24 (quart-Ph*C*–C), phenyl-*C*: 130.88, 130.02, 129.66, 129.27, 126.11, 123.55, cyclohexyl-*C*: 28.81, 28.42, 27.50 ppm.

ESI-MS: [M] = $C_{41}H_{44}BF_3N_6O_3S$; calcd. ([M]–(OTf))$^+$: 619.3715, found: 619.3734, Δ = 3.1 ppm.

Elemental analysis calcd. (%) for $C_{41}H_{44}BF_3N_6O_3S$: C 64.06, H 5.77, N 10.93, S 4.17; found: C 64.07, H 5.79, N 10.97, S 4.44.

[$Ph_2B(diPhtz)_2$](OTf): (H_2L2)OTf

diphenylchloro-borane was synthesized according to literature procedures.[11]

The synthesis was adapted from syntheses of analogous imidazolium compounds.[12]

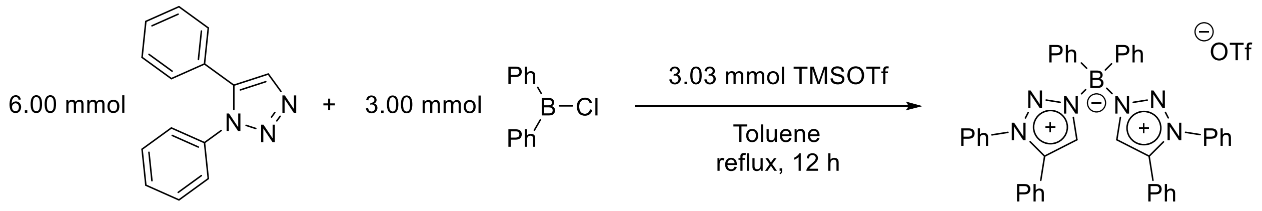

Under an inter atmosphere of argon 1,5-diphenyl-1*H*-1,2,3-triazole (1.3 g, 6.00 mmol) and diphenylchloro-borane (0.6 g, 3.00 mmol) in dry toluene (40 mL) were stirred for 30 min. Trimethylsilyl triflate (0.55 mL, 3.03 mmol) was added and the resulting mixture was refluxed for 12 h under an inert atmosphere. The solvent was removed under reduced pressure and diethyl ether (40 mL) was added. The suspension was kept in an ultrasonic bath for 30 min, and the white precipitant filtered, washed with diethyl ether and dried under vacuum. Recrystallization in toluene and *n*-hexane led to the desired product as a white crystalline solid in yields of 68%.

^{1}H NMR (400 MHz, $CDCl_3$, 20 °C): δ 8.36 (s, 2 H, trz-*H*), 7.58–7.29 (m, 30 H, phenyl-*H*) ppm.

^{13}C NMR (176.1 MHz, $CDCl_3$, 26.9 °C): δ 141.32 (quart-Ph*C*–C), 135.06 (Trz-N–*C*–C), 134.30 (quart-Ph*C*–B), 132.80 (triazole-*C*–H), 131.20 (quart-Ph*C*–N), 128.02, phenyl-*C*: 130.87, 129.85, 129.80, 129.18, 128.02, 126.25, 123.59 ppm.

ESI-MS: [M] = $C_{41}H_{32}BF_3N_6O_3S$; calcd. ([M]–(OTf))$^+$: 607.2776, found: 607.2746, Δ = 4.9 ppm.

Elemental analysis calcd. (%) for $C_{41}H_{32}BF_3N_6O_3S$: C 65.09, H 4.26, N 11.11, S 4.24; found: C 65.15, H 4.28, N 11.12, S 4.52.

1.2.Deprotonation experiments

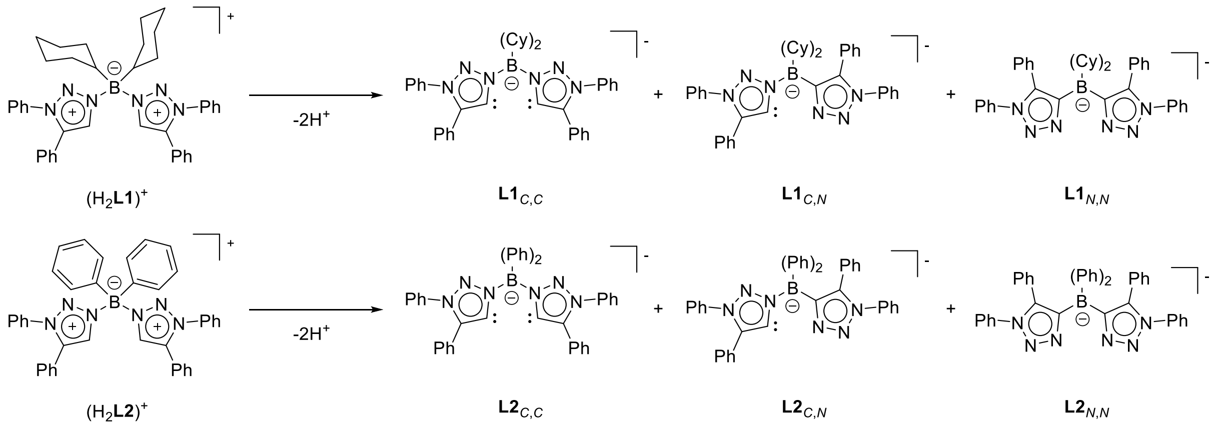

Room temperature monitoring study:

In a dry oxygen-free glovebox ligand (H_2**L1**)OTf (0.063 mmol) and LiTMP (0.125 mmol) were added to a J. Young NMR tube and dissolved in THF-d_8 (0.5 mL). The solution was then monitored by ^{1}H NMR spectroscopy at room temperature. The monitoring study is presented in Figure S1.

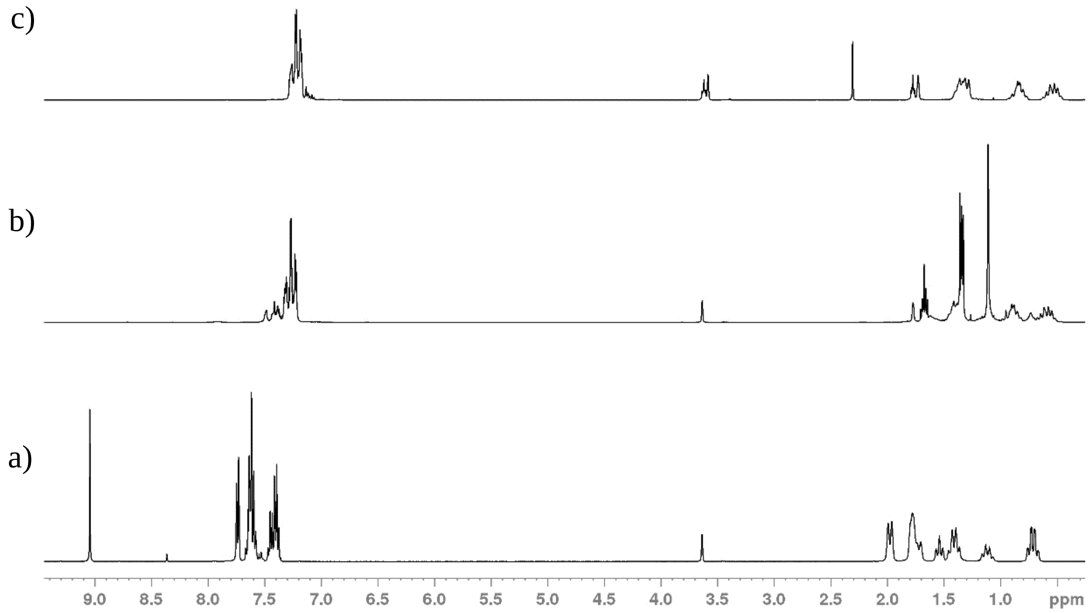

Figure S1: *Reaction monitoring using ^{1}H NMR spectroscopy. a) Starting material, b): 2 equiv. LiTMP, +2 h, rt, c) isolated crystals of [Li**L1**$_{N,N}$]$_2$.*

Low temperature experiment:

The reaction was repeated with the following alterations: As soon as the sample was prepared, it was frozen in a Dewar of liquid nitrogen to prevent reaction. The reaction was warmed to –30 °C and monitored by ^{1}H NMR spectroscopy. The first point to note from the initial spectrum reveals that the deprotonation is instantaneous, as illustrated by the appearance of resonances corresponding to TMPH, and importantly no remaining LiTMP. After 1 hour at this temperature, there is only minimal rearrangement. The sample was next warmed to –20 °C and, once more conversion to the rearranged complex is very slow over a further hour. The sample was then warmed in the spectrometer to 0 °C. At this point the conversion occurs more rapidly. The spectrum after 2 hours at this temperature is shown in Figure S2. Warming to room temperature results in more rapid conversion to the rearranged isomer in ca. 15 minutes.

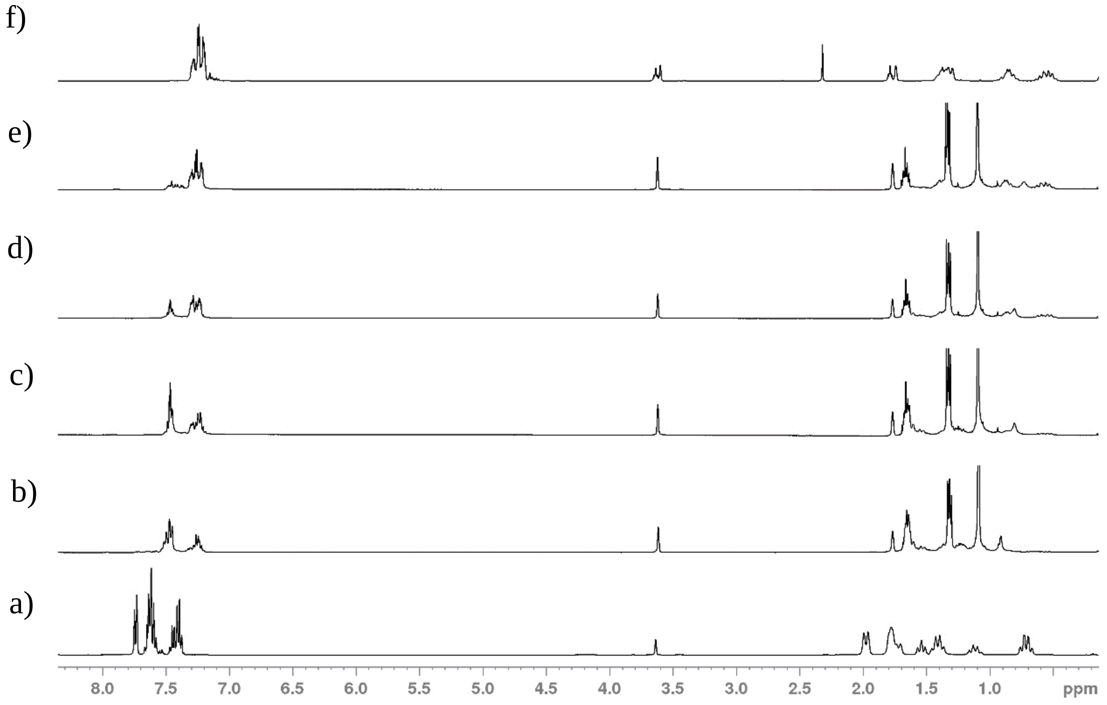

Figure S2: *Low temperature ^{1}H NMR monitoring of deprotonation: a) starting material, b) 2 equiv. LiTMP, +1 h, –30 °C, c) +1 h, –20 °C, d) +2 h, 0 °C, e) +15 min, rt, f) isolated crystals.*

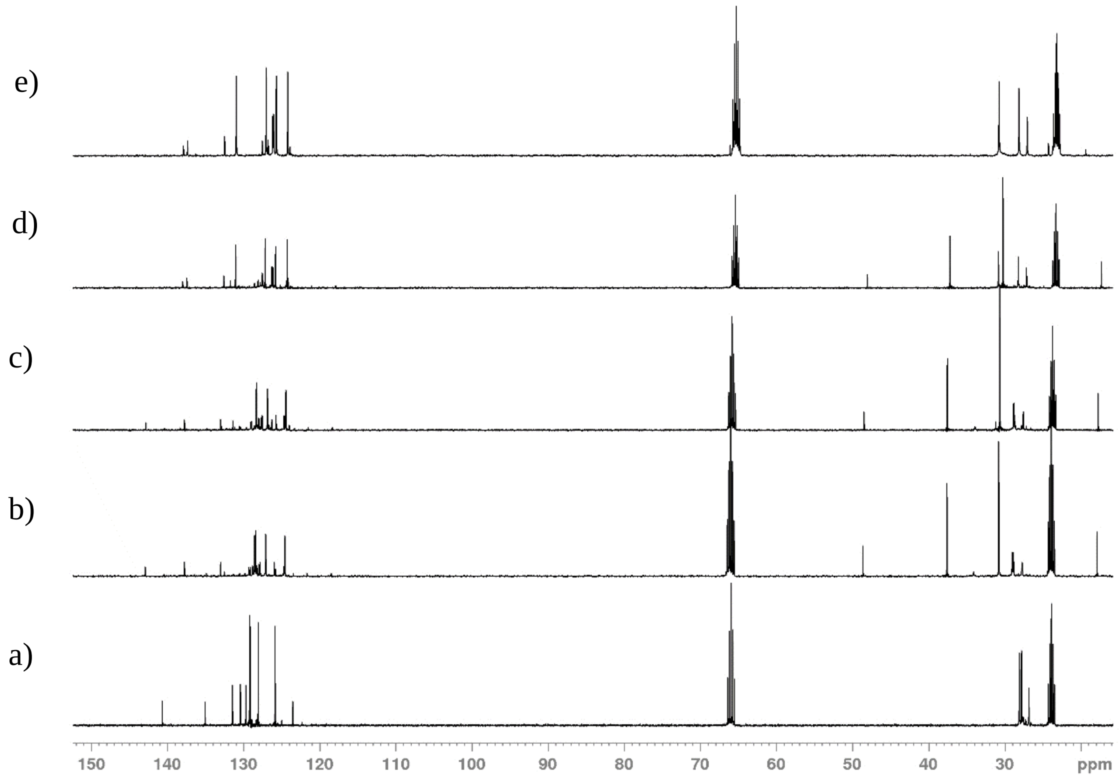

Figure S3: *Low temperature* ^{13}*C NMR monitoring of deprotonation: a) starting material, b) 2 equiv. LiTMP, +1 h, –30 °C, c) 0 °C, d) rt, e) isolated crystals.*

1.3.Complex synthesis

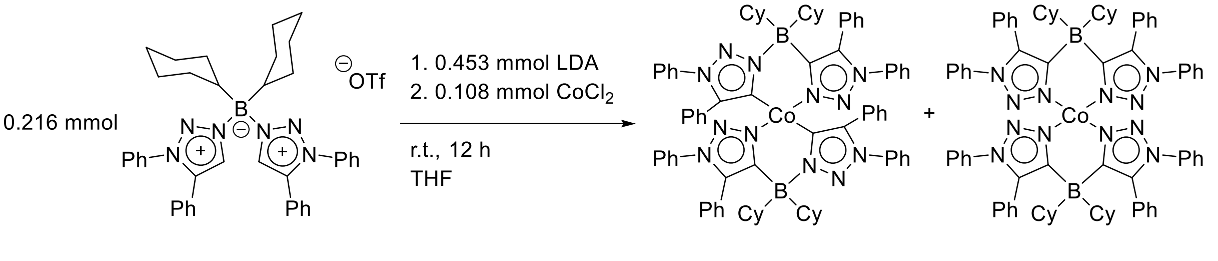

(H$_2$**L1**)OTf [Co(**L1**$_{C,N}$)$_2$] [Co(**L1**$_{N,N}$)$_2$]

Under an inert atmosphere of argon (H$_2$**L1**)OTf (165.8 mg, 0.216 mmol) was dissolved in dry THF (5 mL). Lithium diisopropylamide (0.23 mL, 2.0 M in THF, heptane, ethylbenzene) was added slowly to the solution at room temperature. The mixture was stirred for 30 min, added to dry $CoCl_2$ (16.2 mg, 0.108 mmol) and stirred for additional 12 hours. Then, volatiles were removed under reduced pressure. The solid was re-dissolved in DCM (6 mL) and remaining inorganic salts were filtered through a syringe filter (PTFE, 0.2 µm). The formation of single crystals was obtained by slow diffusion of diethyl ether in the solution. The complexes [Co(**L1**$_{N,N}$)$_2$] and [Co(**L1**$_{C,N}$)$_2$] crystalized as optically inseparable yellow single crystals.

Synthesis [Co(**L1**$_{N,N}$)$_2$]

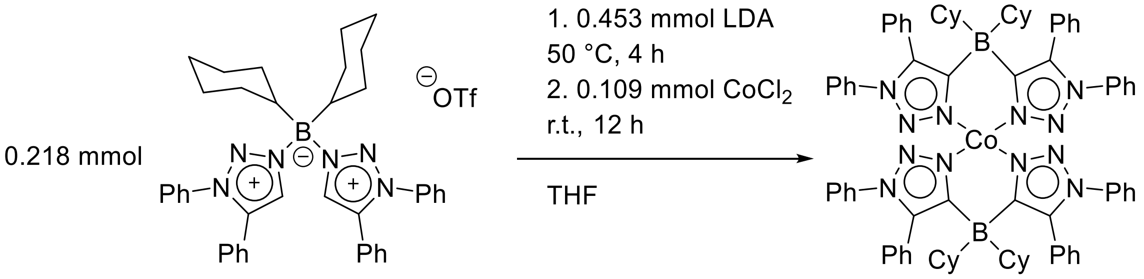

Under an inert atmosphere of argon (H$_2$**L1**)OTf (167.4 mg, 0.218 mmol) was dissolved in dry THF (5 mL). Lithium diisopropylamide (0.23 mL, 2.0 M in THF, heptane, ethylbenzene) was added slowly to the solution at room temperature. The mixture was stirred for 4 hours at 50 °C, added to dry $CoCl_2$ (14.1 mg, 0.109 mmol) and stirred for additional 12 hours. Then, volatiles were removed under reduced pressure. The solid was re-dissolved in DCM (6 mL) and remaining inorganic salts were filtered through a syringe filter (PTFE, 0.2 µm). The formation of single crystals was obtained by slow diffusion of diethyl ether in the solution and the desired complex [Co(**L1**$_{N,N}$)$_2$] crystalized as yellow block crystals in 38.7% yields (54.6 mg).

ESI-MS: [M] = $C_{80}H_{84}B_2CoN_{12}$; calcd. ([M]+Na$^+$)$^+$: 1316.6352, found: 1316.6378, Δ = 2.0 ppm.

Elemental analysis calcd. (%) for $C_{80}H_{84}B_2CoN_{12}$: C 74.25, H 6.54, N12.99; found: C 73.51, H 7.01, N 12.99.

Synthesis [Co(**L2**$_{N,N}$)$_2$]

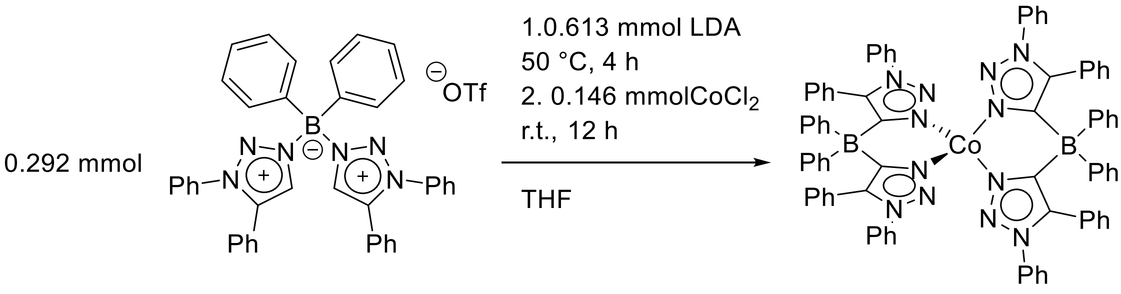

Under an inert atmosphere of argon (H_2**L2**)OTf (220.9 mg, 0.292 mmol) was dissolved in dry THF (8 mL). Lithium diisopropylamide (0.31 mL, 2.0 M in THF, heptane, ethylbenzene) was added slowly to the solution at room temperature. The mixture was stirred for 4 hours at 50 °C, added to dry $CoCl_2$ (19.0 mg, 0.146 mmol) and stirred for additional 12 hours. Then, volatiles were removed under reduced pressure. The solid was re-dissolved in DCM (10 mL) and remaining inorganic salts were filtered through a syringe filter (PTFE, 0.2 µm). The solvent was removed under reduced pressure, toluene was added (6 mL) and the formation of single crystals was obtained by layering *n*-hexane on the solution. The desired complex [Co(**L2**$_{N,N}$)$_2$] crystalized as very fine pink needles in 45.0% yields (84.3 mg).

ESI-MS: [M] = $C_{80}H_{60}B_2CoN_{12}$; calcd. $([M]^+)^+$: 1269.4577, found: 1269.4602; Δ = 2.0 ppm; $([M]+Na^+)^+$: 1292.4474, found: 1292.4502, Δ = 2.1 ppm; $([M]+K^+)^+$: 1308.4214, found: 1308.4281, Δ = 5.1 ppm.

Elemental analysis calcd. (%) for $C_{80}H_{60}B_2CoN_{12}$: C75.66, H 4.76, N 13.23; found: C 75.78, H 4.88, N 13.29.

Synthesis [Co(**L2**$_{N,N}$)$_2$py]

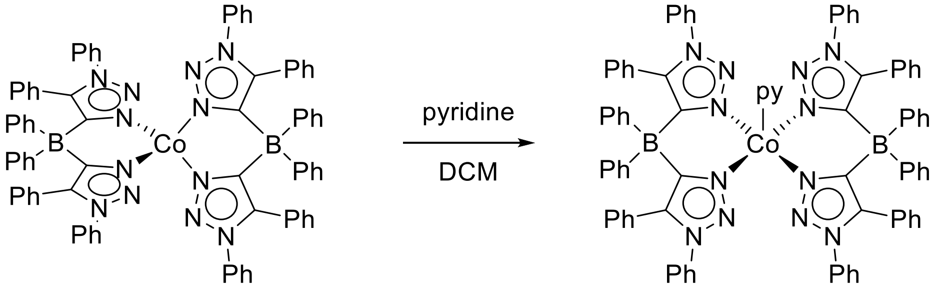

Under an inert atmosphere of argon, [Co(**L2**$_{N,N}$)$_2$] was dissolved in dry and highly degassed DCM. An excess of pyridine was added and the volatiles were removed under reduced pressure. The complex [Co(**L2**$_{N,N}$)$_2$py] could be obtained in quantitative yields. Suitable single crystals were obtained in DCM by slow diffusion of diethyl ether under ambient conditions.

Elemental analysis calcd. (%) for $C_{85}H_{65}B_2CoN_{13}$: C75.68, H 4.86, N 13.50; found: C 75.98, H 5.01, N 13.74.

Synthesis [Li**L1**$_{N,N}$]$_2$

At –50 ºC (H_2**L1**)OTf (0.5 mmol) was added to LiTMP (1.0 mmol) in *n*-hexane (5 mL). THF (5 mL) was added, causing the suspension to pass into solution. This mixture was stirred for a further 2 hours while gradually warming. All volatiles were removed in vacuo, and toluene was added. The white precipitate was separated and the filtrate was collected and all volatiles were removed. The pale-yellow solid was then recrystalised by suspending in *n*-hexane (2 mL) and adding toluene (1.5 mL). At this stage gentle heating was required to completely dissolve the remaining solid. Colourless white crystals then grew at room temperature in 68.0% yield.

^{1}H NMR (400 MHz, THF-d_8, 26.9 °C): δ 7.36–7.14 (m, 20 H), 3.66–3.59 (m, 2 H), 1.82–1.74 (m, 2 H), 1.44–1.18 (m, 10 H), 0.94–0.72 (m, 6 H), 0.65–0.44 (m, 6 H) ppm.

^{13}C NMR (100.1 MHz, THF-d_8, 26.9 °C): δ 140.22, 139.68, 134.80, 133.26, 129.83, 129.34, 129.07, 128.48, 129.36, 128.00, 126.51, 126.20, 32.61, 30.42, 29.34, 26.55, 25.88, 25.68, 25.49, 25.29, 25.08, 21.65, 2.71 ppm.

^{7}Li NMR (155.5 MHz, THF-d_8, 26.9 °C): δ 1.28 ppm.

^{11}B NMR (128.4 MHz, THF-d_8, 26.9 °C): δ –11.47 ppm.

2. NMR Spectra

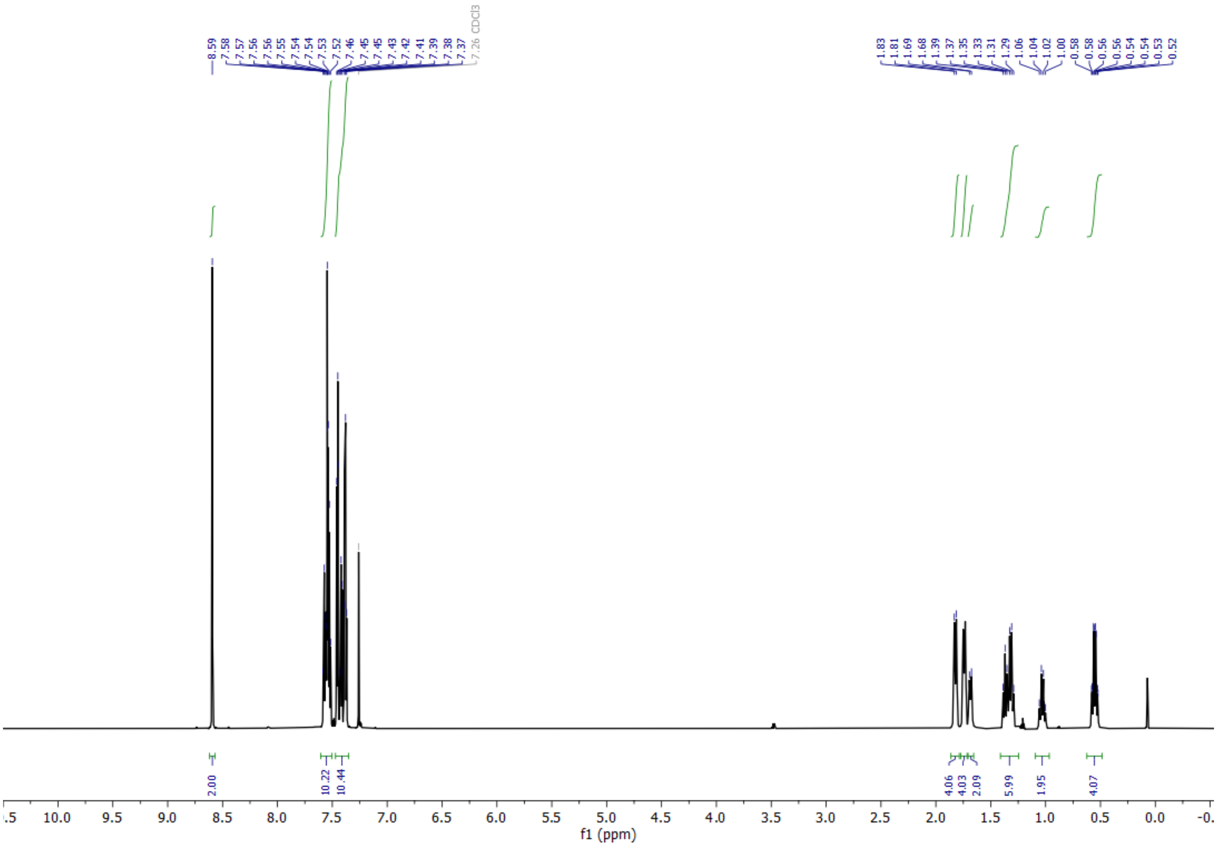

Figure S4*: ^{1}H NMR spectra of (H_2**L1**)OTf in $CDCl_3$.*

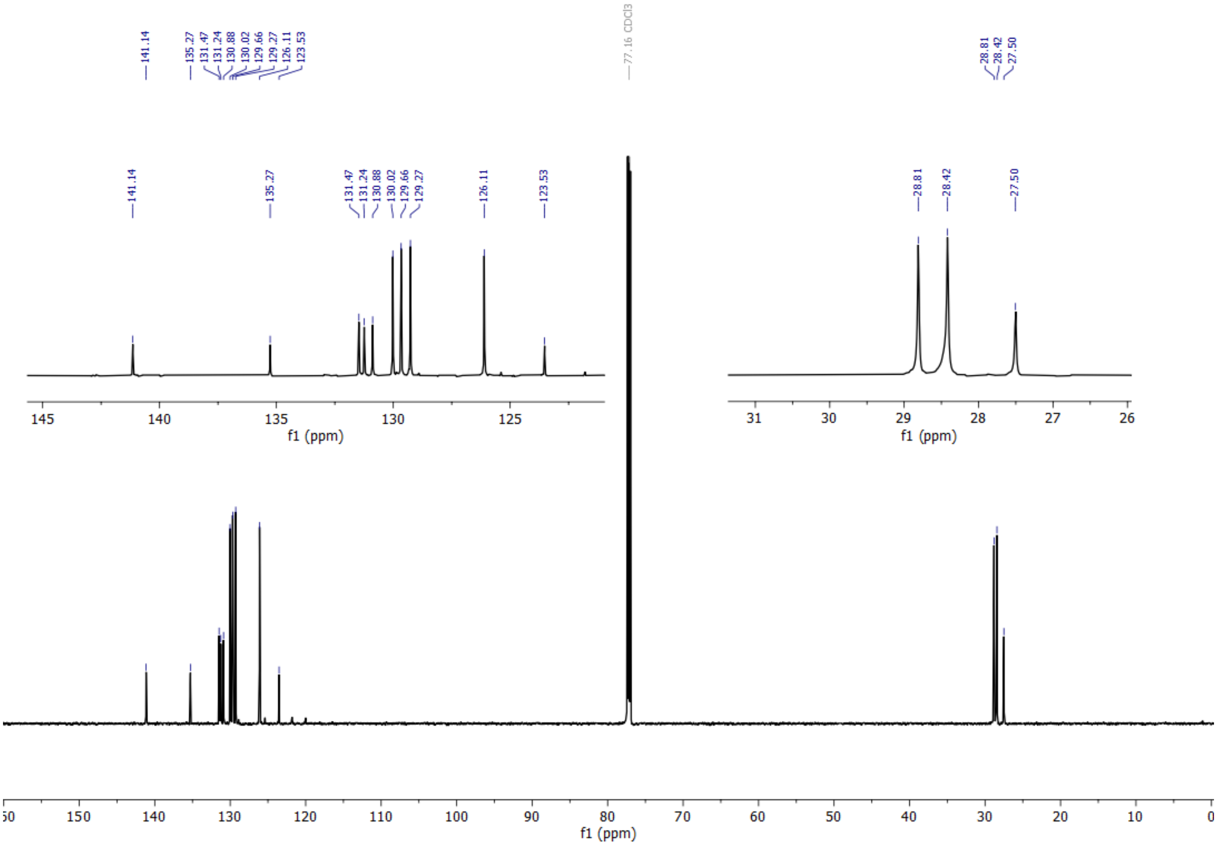

Figure S5*: Proton decoupled ^{13}C NMR spectra of (H_2**L1**)OTf in $CDCl_3$.*

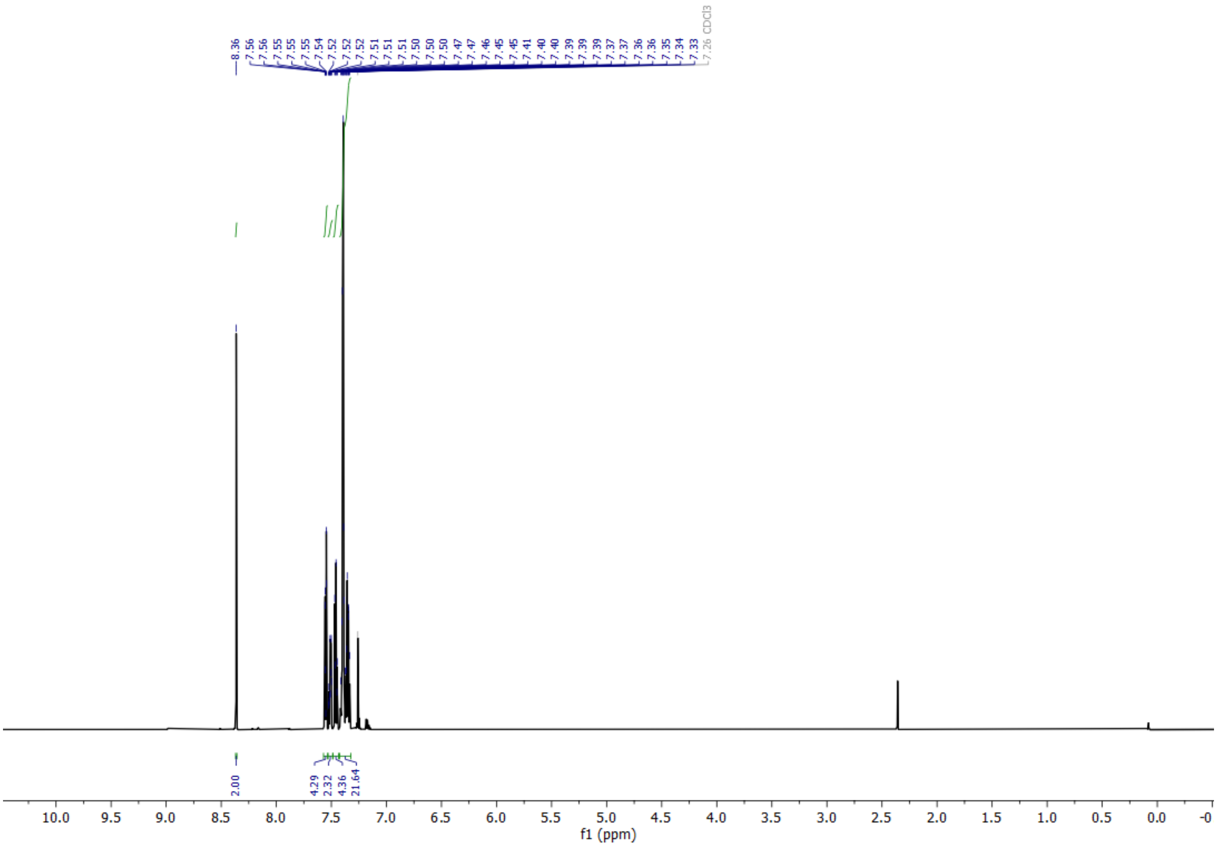

Figure S6: *^{1}H NMR spectra of (H_2**L2**)OTf in $CDCl_3$.*

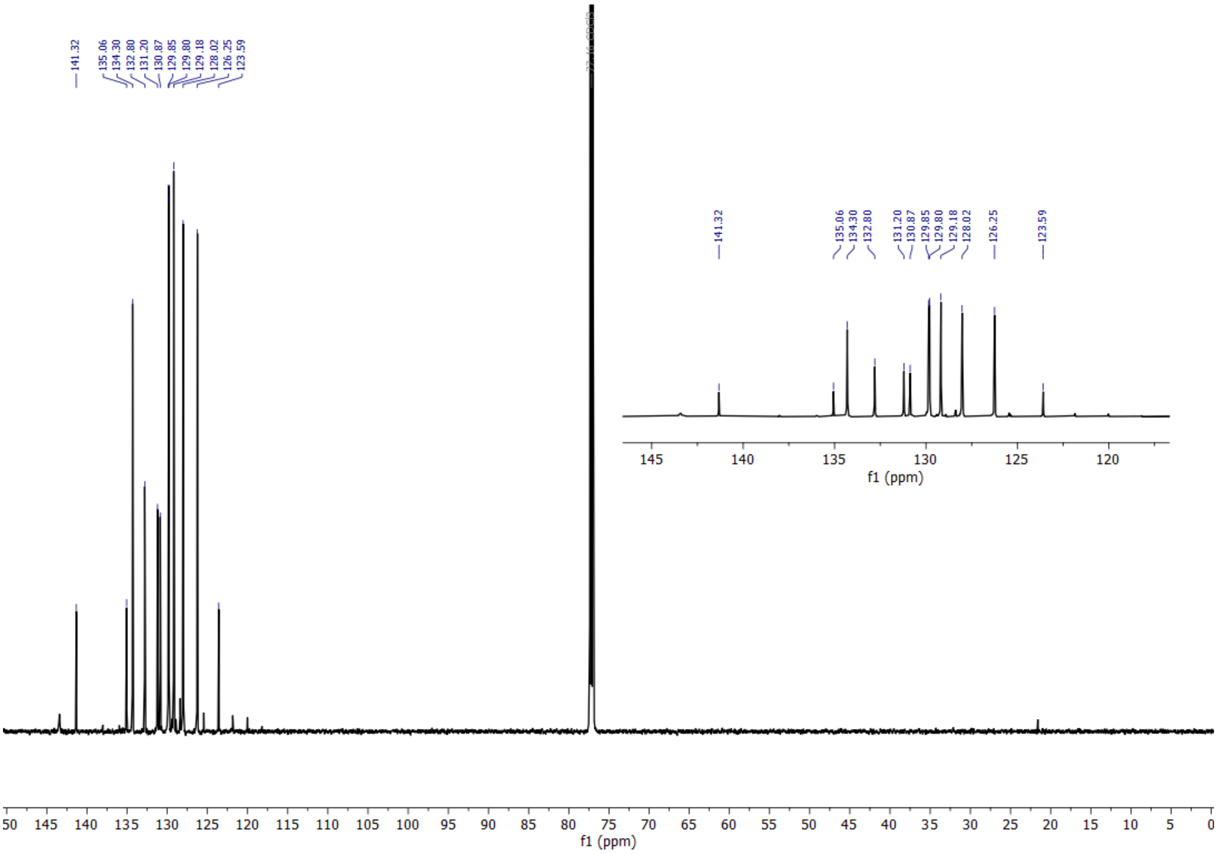

Figure S7: *Proton decoupled ^{13}C NMR spectra of (H_2**L2**)OTf in $CDCl_3$.*

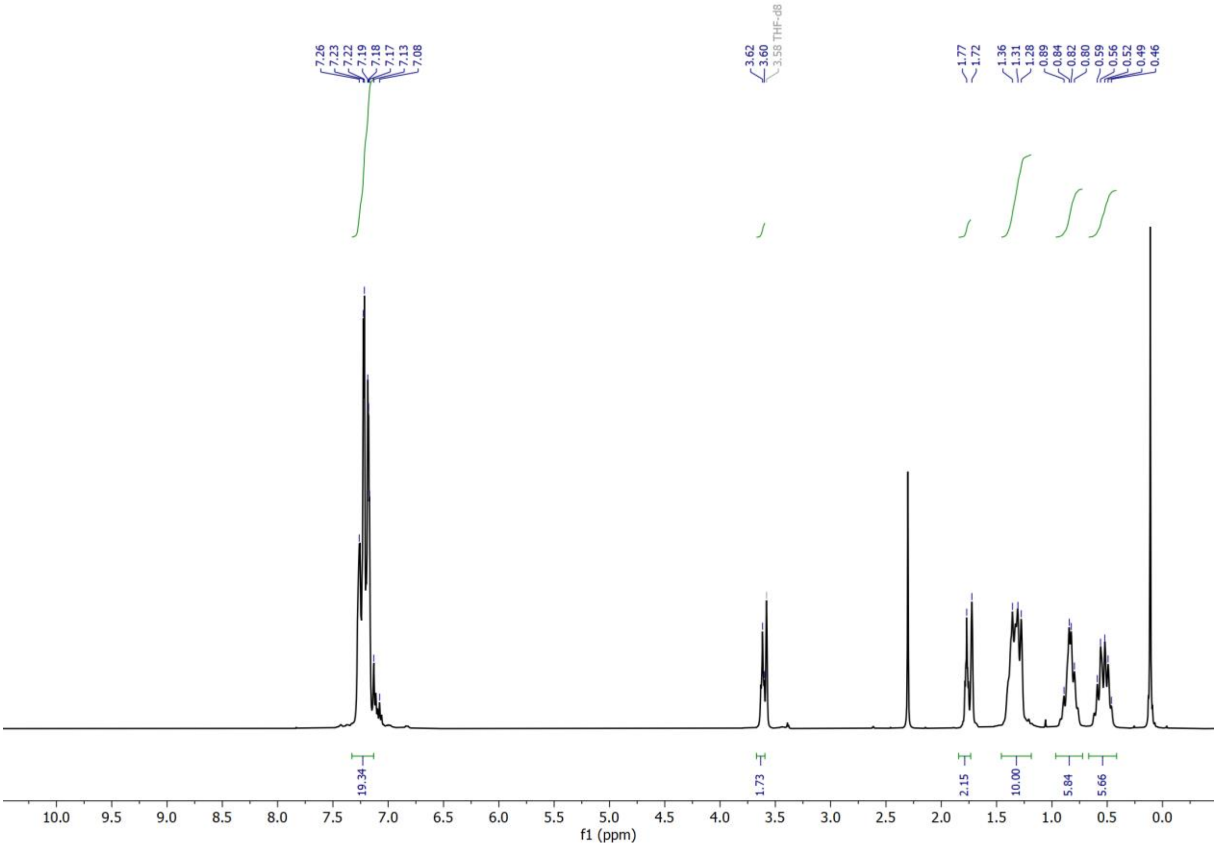

Figure S8:*^{1}H NMR spectra of [Li**L1**$_{N,N}$]$_2$ in THF-d_8.*

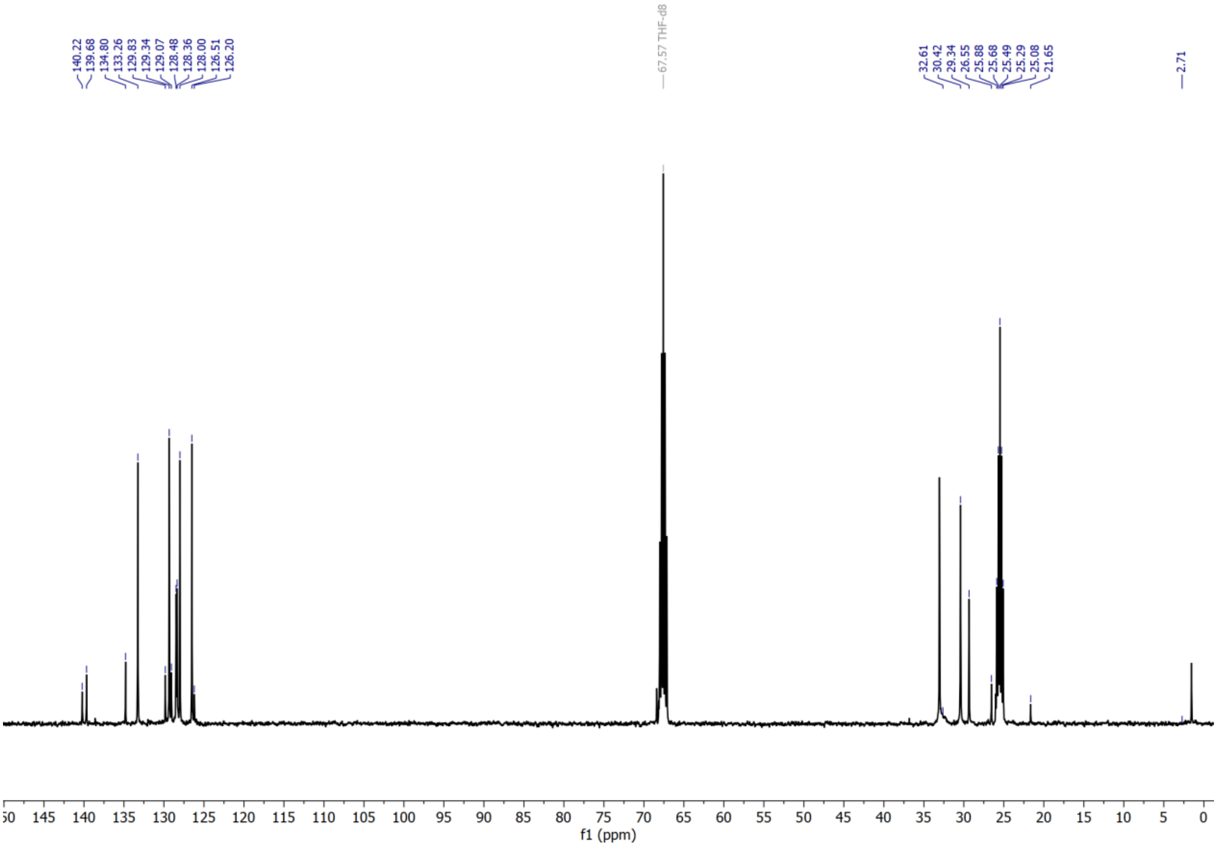

Figure S9: *Proton decoupled ^{13}C NMR spectra of [Li**L1**$_{N,N}$]$_2$ in THF-d_8.*

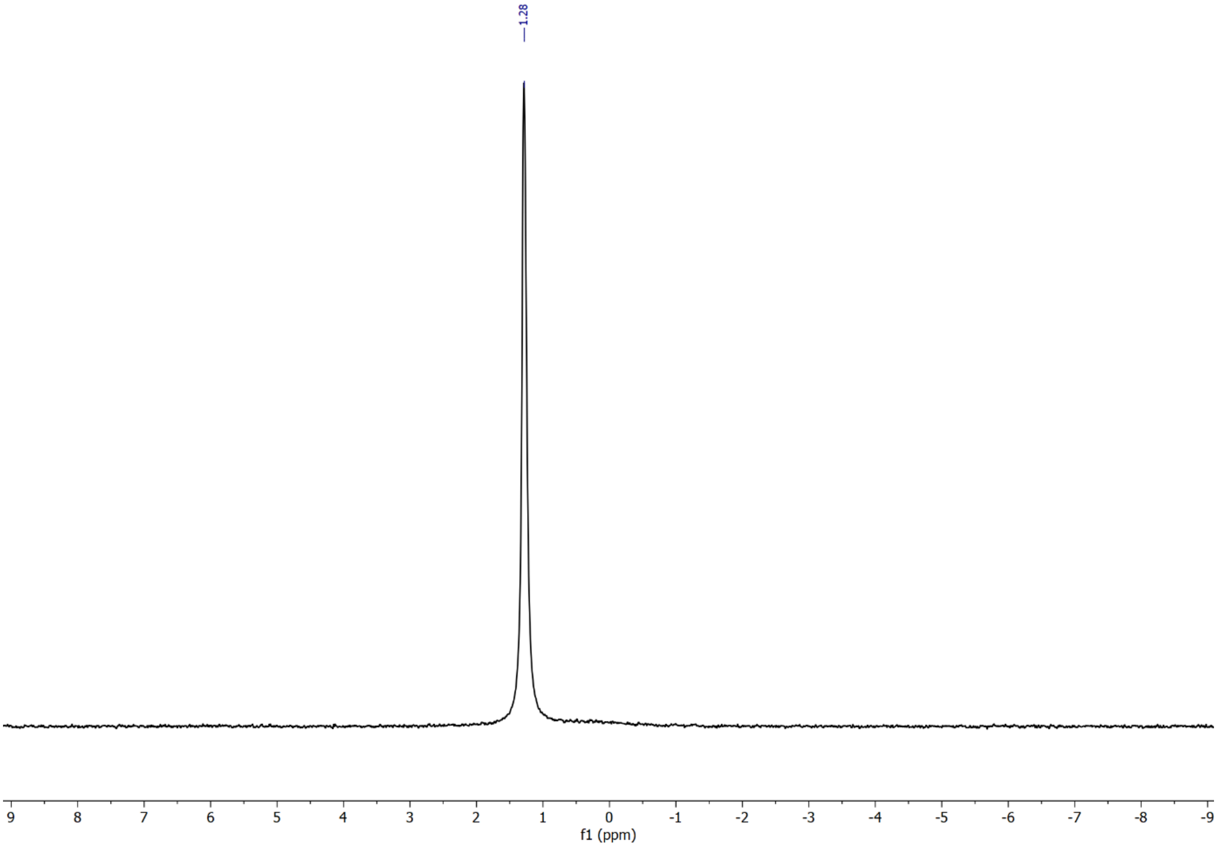

Figure S10: *^{7}Li NMR spectra of [Li**L1**$_{N,N}$]$_2$ in THF-d$_8$.*

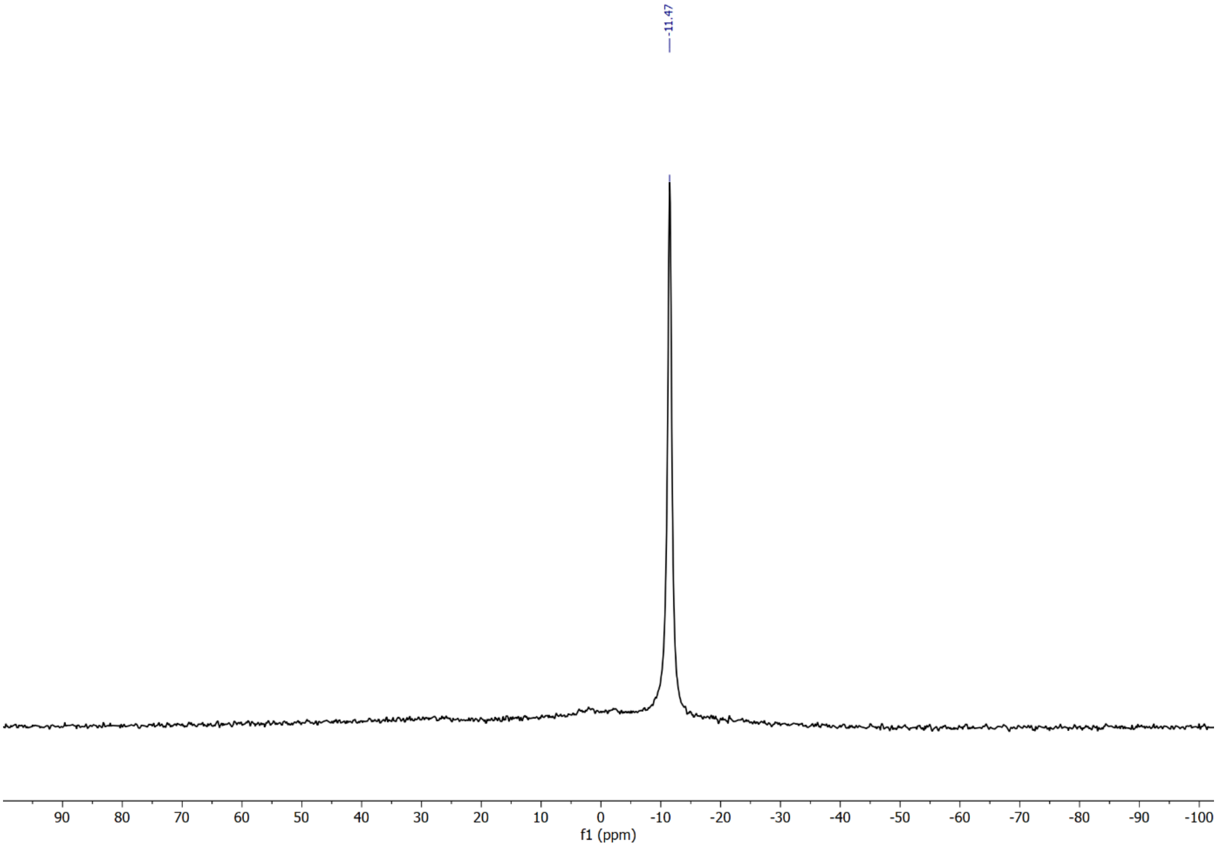

Figure S11: *^{11}B NMR spectra of [Li**L1**$_{N,N}$]$_2$ in THF-d$_8$.*

3. IR Spectroscopy

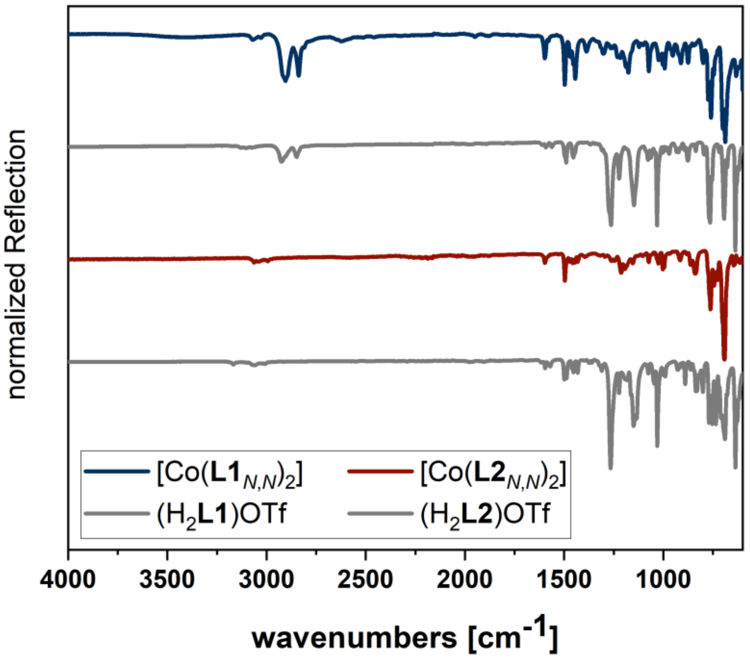

Figure S12: *IR spectra in the solid state*

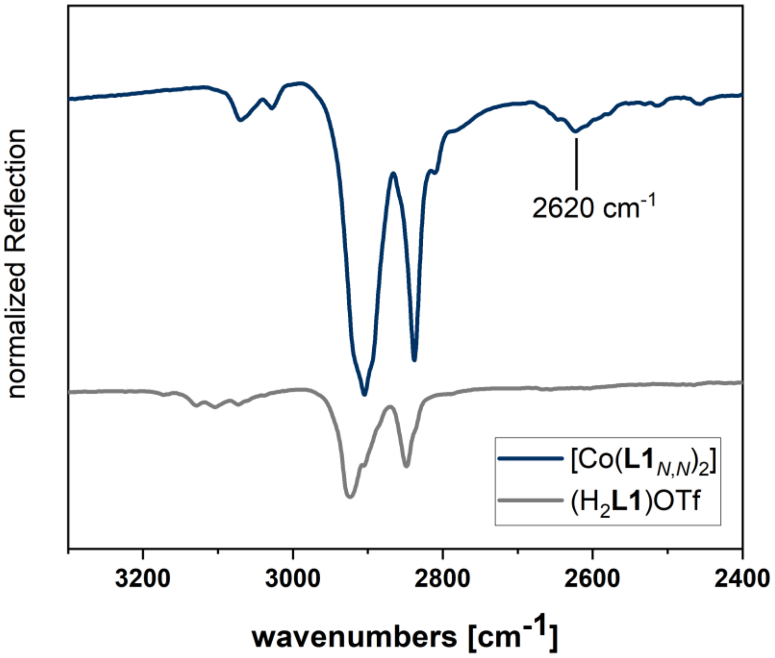

Figure S13: *IR spectra in the solid state*

4. Cyclic Voltammetry

[Co(**L1**$_{N,N}$)$_2$]

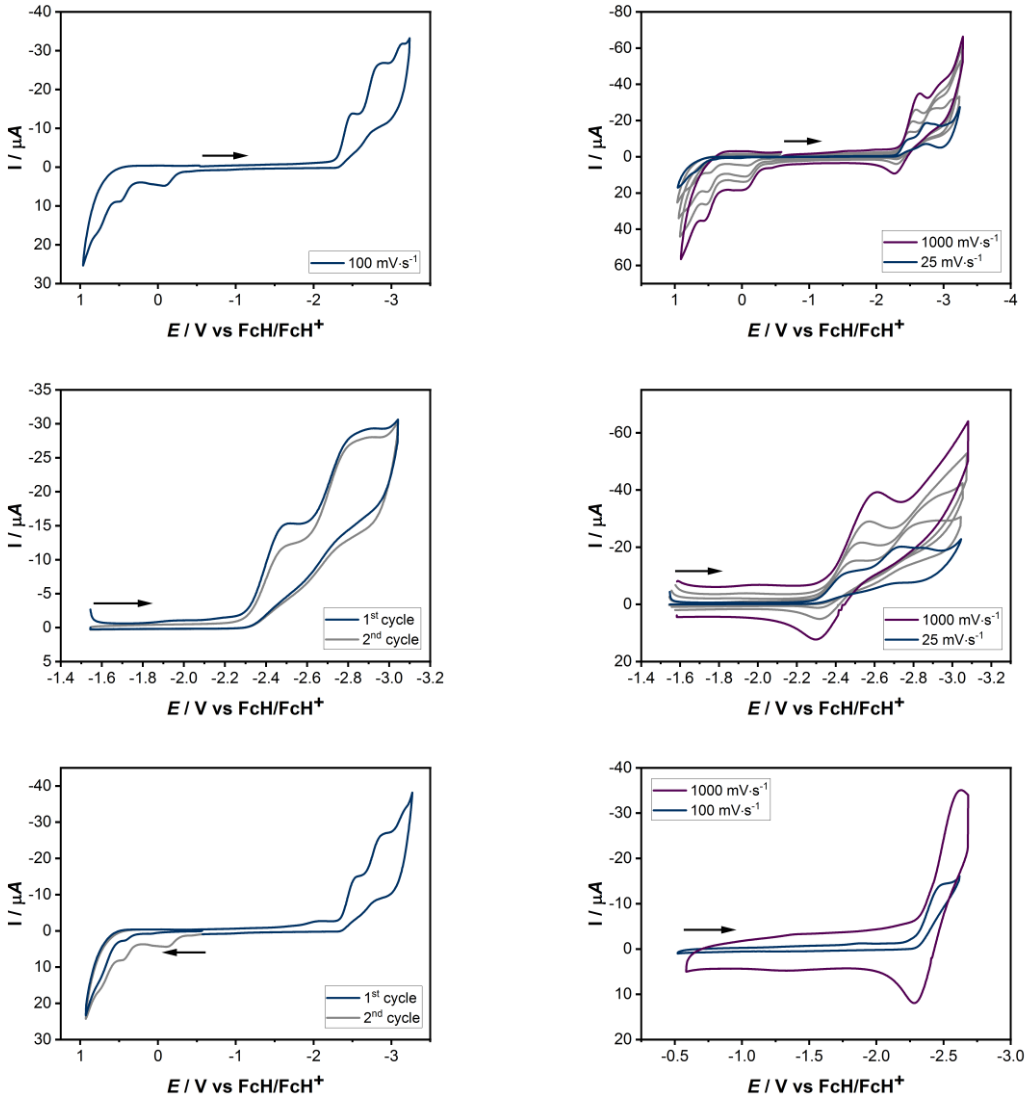

Figure S14: *Cyclic Voltammogram of a 0.1 mM solution of [Co(**L1**$_{N,N}$)$_2$] with 0.1 M NBu$_4$PF$_6$ in THF at left: 100 mV·s^{-1} and right: different scan rates.*

[Co(**L2**$_{N,N}$)$_2$]

Complex [Co(**L2**$_{N,N}$)$_2$] does not show electrochemical processes in the solvent window of dichloromethane and acetonitrile with 0.1 M NBu$_4$PF$_6$ as supporting electrolyte.

5. EPR

Single-crystal EPR spectroscopy: [Co(**L1**$_{N,N}$)$_2$]

The EPR spectra acquired on an oriented single crystal of [Co(**L1**$_{N,N}$)$_2$] show one set of eight well resolved hyperfine lines (I(^{59}Co) = 7/2) arising from a single cobalt(II) centre, which reveals the triclinic $P\bar{1}$ space group of the crystal. Selected single crystal spectra are shown in Figure S15 and the full set of spectra in the three experimental crystal planes are shown in Figure S16.

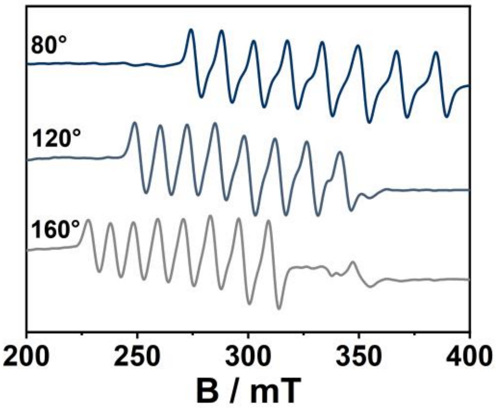

Figure S15: *Single crystal spectra of [Co(**L1**$_{N,N}$)$_2$] in the xy-plane at 80°, 120° and 160°.*

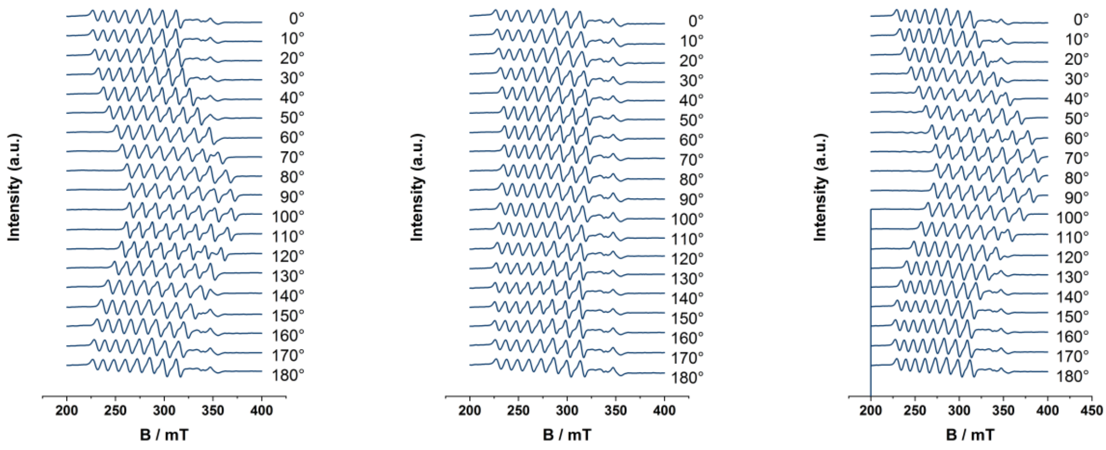

Figure S16: *Full set of single crystal spectra of [Co(**L1**$_{N,N}$)$_2$] in the three experimental planes.*

The well resolved hyperfine splittings and low linewidths indicate that intermolecular magnetic dipolar and exchange interactions are negligible,[13-15] which is congruent with the long Co–Co distances (12.702(1)Å) and with the presence of bulky cyclohexyl and phenyl groups which interact only weakly in the crystal. The central positions of the resonances were automatically determined using Matlab

scripts. From the average (B_0) of the eight resonance positions the *g*-value for each crystal orientation was obtained, and from the average separation *a* (mT) between adjacent resonances, the *K*-factor ($K = ag\mu_B/h$, were μ_B is the Bohr magneton and *h* is Planck's constant) was calculated. Analysis of the angular dependence of the g^2-value and of the g^2K^2-value, ($K = ag\mu_B/h$) allowed us to obtain the **g**-matrix and hyperfine **A**-matrix, following procedures described elsewhere.[16-17]

The **g-** and **A**-matrices were obtained in the experimental *xyz* coordinate system. As the crystal structure belongs to the triclinic $P\bar{1}$ space group, with only one molecule per asymmetric unit, there is one transformation matrix **R** relating the molecular and experimental coordinate frames. Determination of this matrix usually requires knowledge of the morphology of the crystal (*i.e.* assignment of the Miller indices of the major faces of the crystal).

However, given the high symmetry of the square planar coordination around the Co(II) centre, the directions of the eigenvectors of the g-matrix can be *a priori* established. Furthermore, as the CASSCF/NEVPT2 calculations which we performed reproduced the *g*-values remarkably well, the calculated eigenvectors could be used to establish the orientation of the **g**-matrix in the molecular frame.

The angular dependence of the g^2-value in the three orthogonal planes *xy*, *zx* and *zy* is shown in Figure S17.

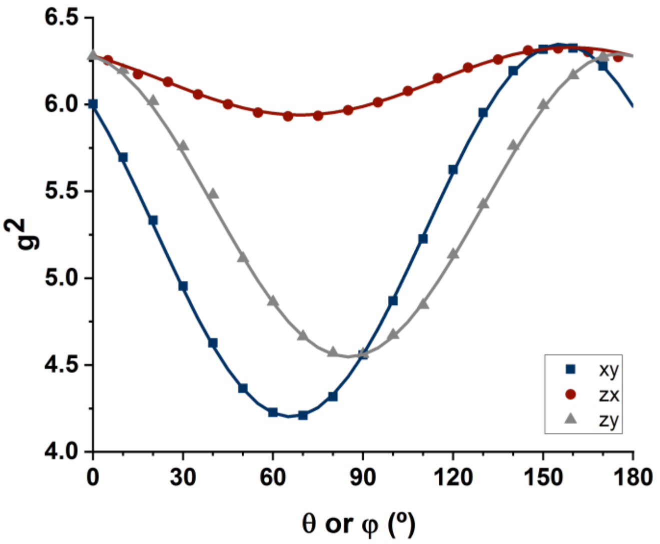

Figure S17: *Angular variation of the crystal* g^2*-value in three orthogonal planes in the experimental xyz coordinate system.*

Table S1. *Eigenvalues and eigenvectors (bold) of the molecular* ***g****- and* ***A****-matrices of the Co(II) ion in compound [Co(L1$_{N,N}$)$_2$].*

g^2_{xx} = 5.989	g^2_{xy} = –0.8
g^2_{yy} = 4.560	g^2_{zx} = –0.13
g^2_{zz} = 6.279	g^2_{zy} = –0.15
g_1 = 2.045(4)	$\mathbf{g}_1$ = [0.409, 0.908, 0.090]
g_2 = 2.502(4)	$\mathbf{g}_2$ = [0.466, –0.293, 0.835]
g_3 = 2.526(5)	$\mathbf{g}_3$ = [0.785, –0.299, –0.542]
A_1 = 460 MHz	$\mathbf{a}_1$ = [0.455, 0.842, –0.288]
A_2 = 405 MHz	$\mathbf{a}_2$ = [0.865, –0.341, 0.368]
A_3 = 423 MHz	$\mathbf{a}_3$ = [–0.212, 0.417, 0.884]

The orientation of the **g**-matrix in the molecular coordinate frame is shown in Figure S18. The g_x and g_y-values are contained in the equatorial CoN_4 plane, forming 27.8º and 24.3º angles with the Co-N bonds. The g_z-value is normal to the equatorial CoN_4 plane.

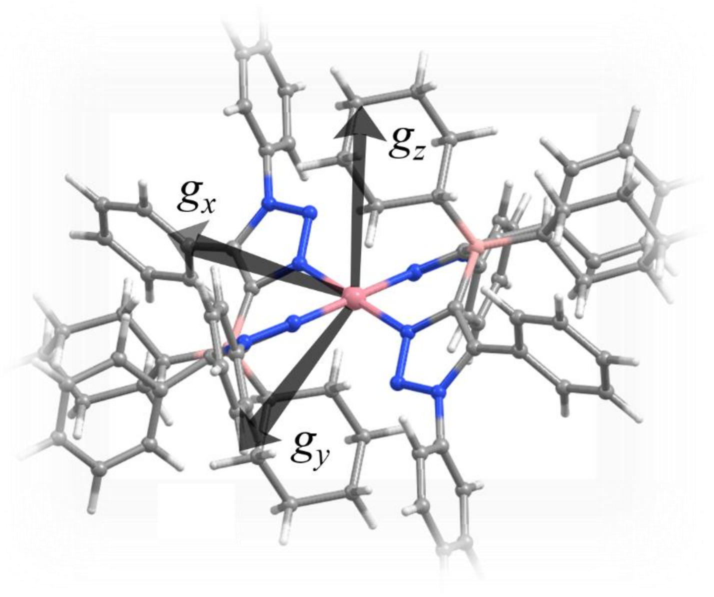

Figure S18: *Orientation of the principal axes of the* ***g****-matrix in the molecular coordinate frame.*

EPR [Co(**L1**$_{N,N}$)$_2$]

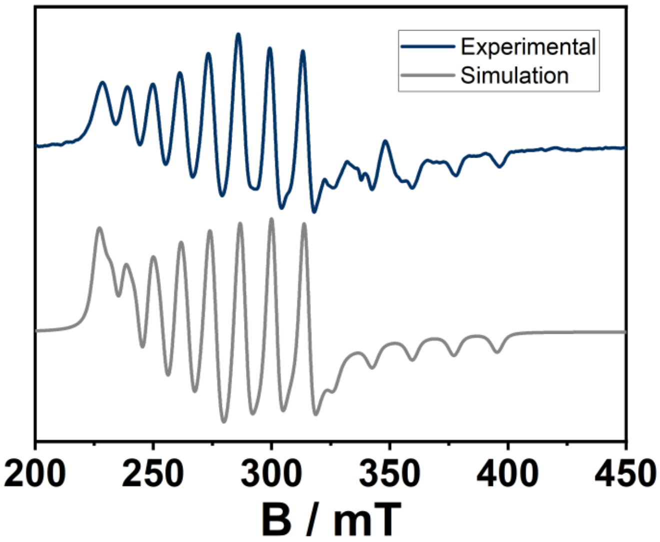

Figure S19: *EPR spectra of [Co(**L1**$_{N,N}$)$_2$] as powder at 100 K. g_x = 2.490, g_y = 2.453, g_z = 1.992, A_x = 437 MHz, A_y = 396 MHz, A_z = 454 MHz.*

EPR [Co(**L2**$_{N,N}$)$_2$]

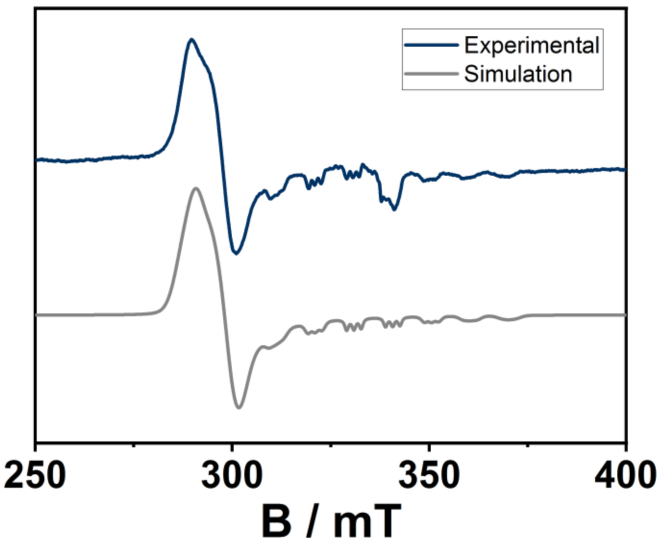

Figure S20: *EPR spectra of [Co(**L2**$_{N,N}$)$_2$] in DCM with an excess of **pyridine** at 100 K.*

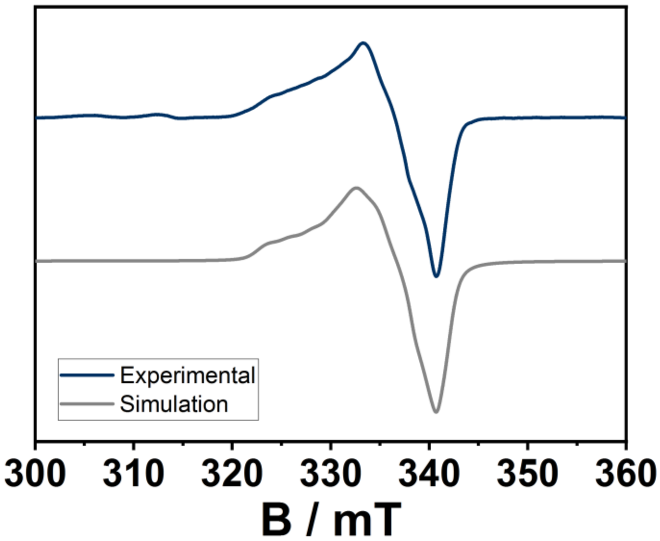

Figure S21: *EPR spectra of [Co(**L2**$_{N,N}$)$_2$] in DCM in the presence of **O_2** at 100 K.*

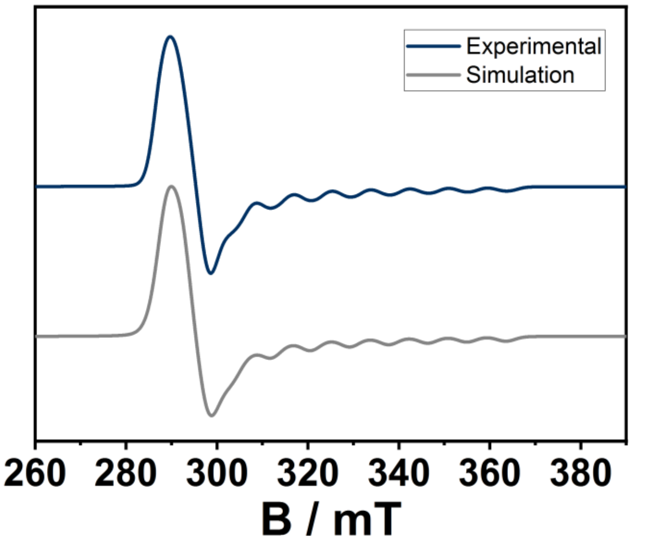

Figure S22: *EPR spectra of [Co(**L2**$_{N,N}$)$_2$py] as a powder at 100K.*

EPR results

Table S2: *Experimentally determined EPR values at 100 K.*

	g_x	g_y	g_z	A_x	A_y	A_z	^{14}N: A_z
[Co(**L1**$_{N,N}$)$_2$] (powder)	2.490	2.453	1.992	437	396	454	
[Co(**L2**$_{N,N}$)$_2$] (DCM + Py)	2.298	2.254	2.015	19.7	5.5	279	52
[Co(**L2**$_{N,N}$)$_2$py] (powder)	2.298	2.290	2.028	17.5	19.2	243	
[Co(**L2**$_{N,N}$)$_2$] (DCM + O_2)	1.993	2.024	2.040	9	13	65	

6. Electronic Structure Calculations

In order to correlate the results from EPR experiments with the electronic structure and ligand field energetics of the different cobalt complexes we performed complete-active space self-consistent field (CASSCF) calculations, with multi-reference perturbation theory (NEVPT2) to take into account dynamical correlation effects.

All calculations were performed with the ORCA program package, version 4.0.1.2.[18] Single-crystal X-ray diffraction derived structures were used without optimization. For the truncated complex **[Co(L1$_{N,N}$)$_2$]^{Tr}** the apical CH_2 groups were removed and capping H atoms were added manually. Multireference complete active space self-consistent field (CASSCF) calculations,[19-20] together with the N-electron valence perturbation theory of order 2 (NEVPT2)[21-23] were performed on the structures of compounds [Co(**L1**$_{N,N}$)$_2$], [Co(**L1**$_{N,N}$)$_2$]^{Tr} and [Co(**L2**$_{N,N}$)$_2$py]. The active space in all cases was composed of 7 electrons distributed in 5 cobalt 3*d* orbitals (CAS(7,5)). For compounds [Co(**L1**$_{N,N}$)$_2$], [Co(**L1**$_{N,N}$)$_2$]^{Tr}, 10 quartet and 40 doublet roots were included in the state-averaged CASSCF calculation, while for [Co(**L2**$_{N,N}$)$_2$py], based on preliminary calculations and taking into account the increase in the crystal field strength due to the pyridine ligand, we performed the calculation including 30 doublet roots. Ahlrichs type basis sets def2-SVP were used on all atoms except Co, for which def2-TZVP basis sets were used.[24] The resolution-of-the-identity (RI) approximation[25-31] with matching basis sets,[32-34] as well as the RIJCOSX approximation (combination of RI and chain-of-spheres algorithm for exchange integrals) were used to reduce the time of calculations. We also performed DFT calculations using the PBE functional[35] with def2-TZVP basis set on all atoms. Visualization of structures, orbitals and spin densities were done using the program Chemcraft.[36]

We found that for all studied complexes the low spin multiplicity was the ground state, and that considering 20-30 doublet roots were enough to predict *g*-values in great agreement with experiment.

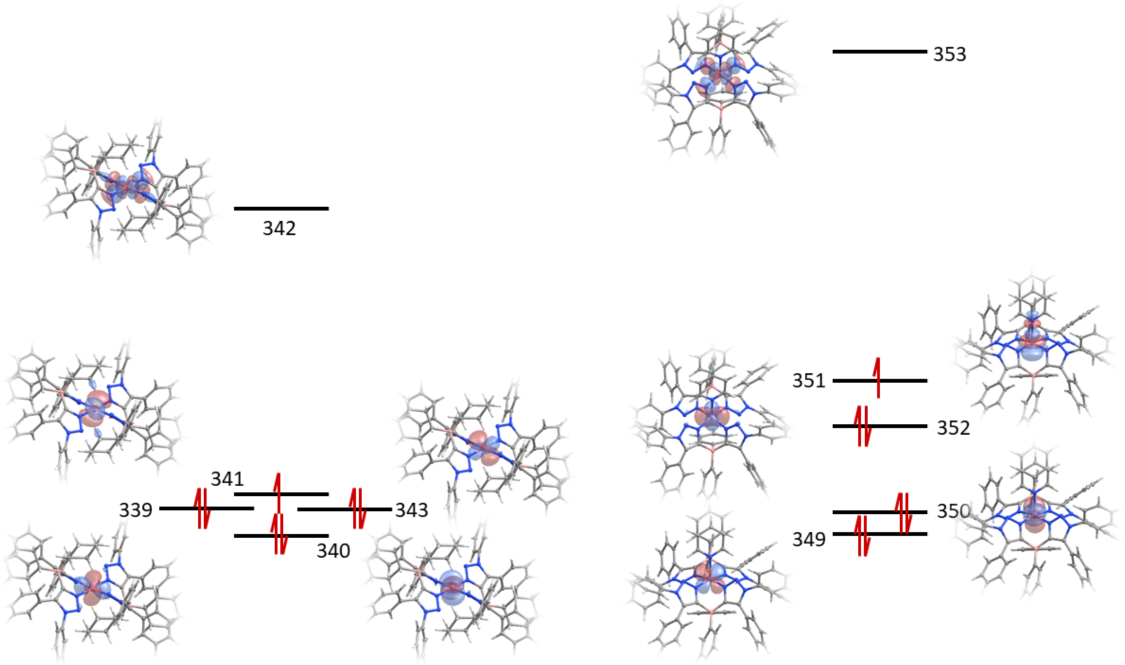

Figure S23: *Orbital energy diagram for the 3d orbitals of [Co(**L1**$_{N,N}$)$_2$] and [Co(**L2**$_{N,N}$)$_2$py].*

Table S3 shows the energies and dominant configuration compositions of the first roots of [Co(**L1**$_{N,N}$)$_2$] calculated taking into account 40 doublet and 10 quartet roots. The orbitals are those depicted in the left panel of Figure S23. These orbitals are mixtures of the canonical *d*-orbitals and roughly correspond to $(x^2\text{-}y^2)(xz)(z^2)(xy)(yz)$, in the order considered in the calculation. The x and y directions are defined between the Co–N bonds.

Table S3: *Lowest energy roots for the doublet and quartet states of **[Co(L1$_{N,N}$)$_2$]**, **[Co(L1$_{N,N}$)$_2$]^{Tr}**, and **[Co(L2$_{N,N}$)$_2$py]**. The composition of the roots in terms of the most important Configuration State functions (CSFs) are indicated, as well as the d-orbital ordering for each calculation.*

**I**		**[Co(L1$_{N,N}$)$_2$]**		
Multiplicity	Roots	Dominant configurations	CASSCF Energies (cm^{-1})	NEVPT2 Energies (cm^{-1})
2	0	0.60[22102]+0.28[22201]	0	0
	1	0.83[21202]	4877.3	5434.8
	2	0.56[22201]+0.20[22102]	4953.3	5549.0
	3	0.44[12202]+0.31[21211]	13548.1	16386.1
4	0	0.67[22111]+0.15[12112]+0.13[12211]	–2667.9	4287.9
	1	0.46[21211]+0.38[11212]+0.13[21112]	–2366.8	4644.6
	2	0.66[12112]+0.26[22111]	–1008.7	6419.9
		Orbital ordering		
		xy, xz,[a] z^2,[a] x^2-y^2, yz[a]		

		$[Co(L1_{N,N})_2]^{Tr}$		
Multiplicity	Roots	Dominant configurations	CASSCF Energies (cm^{-1})	NEVPT2 Energies (cm^{-1})
2	0	0.65[02221]+0.28[01222]	0	0
	1	0.59[01222]+0.18[02221]+0.13[02122]	3131.0	2846.2
	2	0.88[02212]	3245.5	3042.9
	3	0.45[02122]+0.26[11212]	11815.2	13673.3
4	0	0.30[11212]+0.22[11122]+0.15[12211]	-2551.0	4283.9
	1	0.44[11221]+0.31[12121]	-2463.3	4302.1
	2	0.30[11212]+0.30[12121]+0.23[11221]	-1181.8	6006.0
		Orbital ordering		
		x^2-y^2, xz,[a] xy, yz,[a] $z^{2,a}$		

		$[Co(L2_{N,N})_2py]$		
Multiplicity	Roots	Dominant configurations	CASSCF Energies (cm^{-1})	NEVPT2 Energies (cm^{-1})
2	0	0.94[22120]	0	0
	1	0.80[21220]+0.14[12121]	7239.4	9346.6
	2	0.81[12220]+0.13[21121]	7424.8	9560.0
	3	0.84[22021]+0.08[22111]	10980.7	14663.6
		Orbital ordering		
		xz, yz, z^2, xy, x^2-y^2		

Table S4*: Comparison of experimental and calculated* ***g****-matrix components for* $[Co(L1_{N,N})_2]$ *and* $[Co(L2_{N,N})_2py]$*, as well as calculated components for* $[Co(L1_{N,N})_2]^{Tr}$*. Calculations were performed at the CASSCF/NEVPT2 level with an active space of 7 electrons in 5 3d orbitals.*

	$[Co(L1_{N,N})_2]$ Experimental (Single Crystal EPR)	$[Co(L1_{N,N})_2]$ (CASSCF/ NEVPT2)	$[Co(L1_{N,N})_2]^{Tr}$ (CASSCF/ NEVPT2)	$[Co(L2_{N,N})_2py]$ (CASSCF/ NEVPT2)	$[Co(L2_{N,N})_2py]$ Experimental (powder EPR)
g_x	2.502	2.512	2.815	2.296	2.298
g_y	2.526	2.529	2.891	2.319	2.290
g_z	2.045	2.004	1.965	1.994	2.027

The magnetic and EPR properties of low spin cobalt(II) complexes have long been of interest to coordination chemists. One of the earliest theoretical analyses in this field were performed by Maki et al.[37] and later by McGarvey.[38] The latter author's study focused on low spin Co(II) complexes of tetragonal and orthorhombic symmetry and treated the following alternatives: I) the ground state configuration contains an unpaired electron on a linear combination of the $d(x^2\text{-}y^2)$ and $d(z^2)$ orbitals ($ad(x^2\text{-}y^2) + bd(z^2)$); II) the unpaired electron is in a $d(yz)$ orbital and it applies to the case where the *xz*-plane is a mirror plane in C_{2v} symmetry. In alternative (I) there are two extreme cases, where $a = 1$ (unpaired electron in the $d(x^2\text{-}y^2)$ orbital) and $b = 1$ (unpaired electron in the $d(z^2)$ orbital). The latter case (unpaired electron on the $d(z^2)$ orbital) is the one relevant to the compounds studied in this work. Equations (27-30) in McGarvey's work apply to this case and are the most relevant for our work. These equations are written using coefficients c_i which correspond to the ratio between the spin-orbit coupling constant ξ and energy differences Δ_{ij} between the ground state and excited configurations, and they include couplings of the ground state (S = 1/2) with M_S = ±1/2 wavefunctions from the excited S = 3/2 configurations. We here reproduce those equations replacing the original coefficients c_i by their expressions in terms of ξ/Δ_{ij}. We also group all the terms involving double excitations into a single approximate expression $\xi/\Delta(1/2\text{-}3/2)$, whose large contribution is from the third excited doublet root.

$$g_{||} = g_e - 3\frac{\xi^2}{\left(E(d_{xz}) - E(d_{z^2})\right)^2} + 2\frac{\xi^2}{\Delta^2_{\frac{1}{2}\to\frac{3}{2}}}$$

$$g_{\perp} = g_e + 6\frac{\xi}{\left(E(d_{xz}) - E(d_{z^2})\right)} - 6\frac{\xi^2}{\left(E(d_{xz}) - E(d_{z^2})\right)^2} + 2\frac{\xi^2}{\Delta^2_{\frac{1}{2}\to\frac{3}{2}}}$$

The terms $E(d_{xz})$ and $E(d_{z2})$ do not mean the energies of the orbitals, but the energies of the configurations where these orbitals contain the unpaired electron. Using the energies of the roots from the calculations in Table S3, and considering a spin orbit coupling constant of ~500 cm^{-1} (free ion value) we can see that for [Co(**L1**$_{N,N}$)$_2$] the terms quadratic in ξ/Δ_{ij} have a value lesser than 0.015, while the linear term has a value of ~0.092. Therefore, we can estimate the following *g*-values for [Co(**L1**$_{N,N}$)$_2$]: $g_{||}$ = 2.0023 and $g_{\perp}$ = 2.53, which are remarkably in agreement with the experimental values given the approximations we have made in applying McGarvey's equations. For [Co(**L2**$_{N,N}$)$_2$py], McGarvey's equations predict $g_{||}$ = 1.9918 and $g_{\perp}$ = 2.310, in great agreement with the experimental values. The purpose of these ligand field calculations is not to provide exact *g*-values (as this can now easily be done with CASSCF/NEVPT2 calculations) but to provide a qualitative idea of which orbital couplings have a larger importance on the g-values and which energies are reasonable. The conclusion from this analysis is that the first three excited doublet roots are enough to qualitatively explain the g-values in these type of tetragonal low spin Co(II) systems, and therefore quite accurate magneto-structural correlations could be obtained from a combination of EPR spectroscopy and CASSCF calculations.

Calculated IR Spectrum

We performed a single-point Density Functional Theory (DFT) calculation on the structure of **[Co(L1$_{N,N}$)$_2$]**, followed by a frequency calculation, in order to obtain the calculated IR spectrum. Due to the large size of the molecule and the computationally expensive nature of the frequency calculation, we used the pure-GGA PBE functional, together with a def2-TZVP basis set. Since IR spectra were acquired in the solid state, and DFT optimization would not take into account the crystal packing, the structure was not optimized. This factor, together with the relatively inaccurate theoretical level, results in only a qualitative reproduction of the experimental IR spectra (depicted in Figure S13). However it can be seen that the C-H stretching frequencies associated with the cyclohexyl substituent atoms not directly interacting with the Co(II) ion occur in the 2970-3100 cm^{-1} region, while the cyclohexyl C-H stretching frequencies directly involved in the agostic interaction are red-shifted and appear at 2916-2918 and 2953-2954 cm^{-1}. This qualitative behavior is in line with the experimental findings and support the assignment of the experimental band around 2620 cm^{-1} to C-H stretching frequency involving the CH_2 groups presenting agostic interactions.

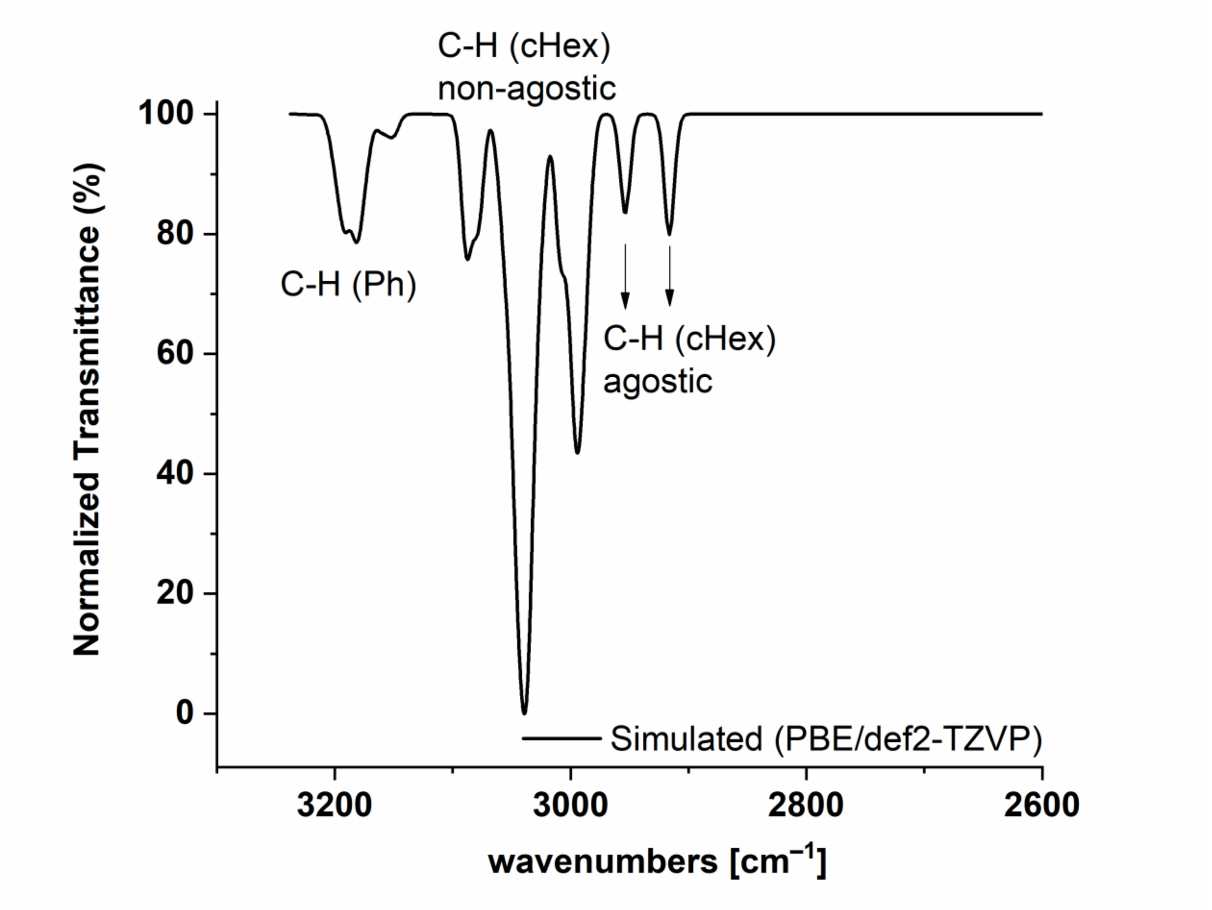

Figure S24: *Simulated IR spectrum of **[Co(L1$_{N,N}$)$_2$]** in the 3250-2600 cm^{-1} region.*

7. Crystal Structures & Crystallographic Data

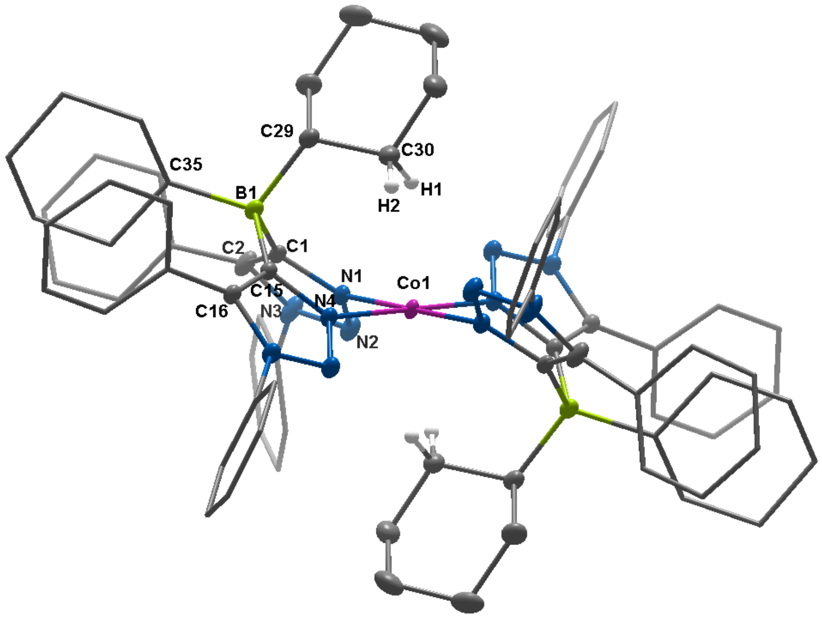

Figure S25: *ORTEP representation of [Co(**L1**$_{N,N}$)$_2$]: ellipsoids drawn at 50% probability. Solvent molecules and H-atoms omitted for clarity.*

Table S5: *Selected bond length and bond angles of [Co(**L1**$_{N,N}$)$_2$].*

Atom	Bond length (Å)	Atom	Bond angle (°)
Co1–N1	1.907(1)	N1–Co1–N4	86.63(5)
Co1–N4	1.918(1)	N1–Co1–N4	93.37(5)
Co1–H1	2.31(3)	C1–N1–N2	113.3(1)
Co1–H2	2.69(2)	N1–N2–N3	105.0(1)
N1–N2	1.317(2)	N2–N3–C2	111.8(1)
N2–N3	1.331(2)	N3–C2–C1	105.9(1)
C2–N3	1.373(2)	C35–B1–C29	110.5(1)
C1–C2	1.392(2)	C29–B1–C1	109.9(1)
N1–C1	1.378(2)	C35–B1–C15	115.5(1)
B1–C1	1.658(2)	C29–B1–C15	109.0(1)
B1–C15	1.662(2)	C1–B1–C15	104.0(1)
B1–C29	1.658(2)		
B1–C35	1.649(2)		

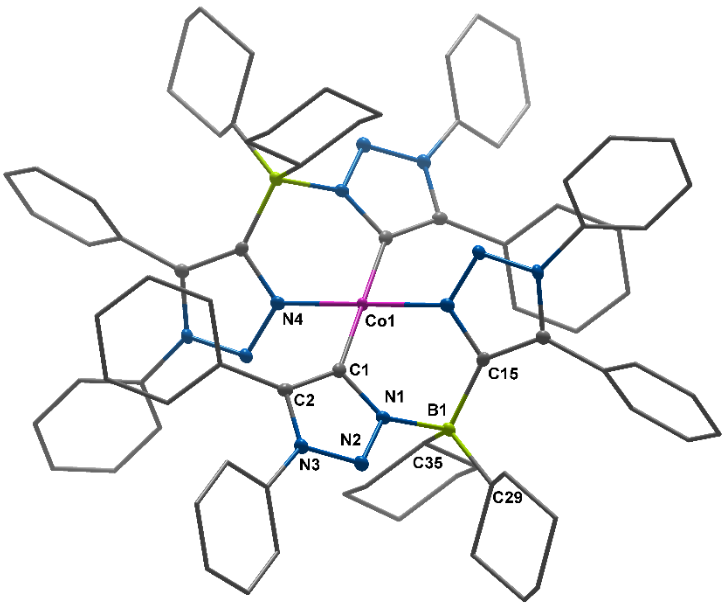

Figure S26: *ORTEP representation of [Co(**L1**$_{C,N}$)$_2$]: ellipsoids drawn at 50% probability. Solvent molecules and H-atoms omitted for clarity.*

Table S6: *Selected bond length and bond angles of [Co(**L1**$_{C,N}$)$_2$].*

Atom	Bong length (Å)	Atom	Bond angle (°)
Co1–N4	1.916(2)	N4–Co1–C1	91.54(8)
Co1–C1	1.974(2)	N4–Co1–C1	88.46(8)
C1–C2	1.394(3)	N3–C2–C1	106.5(2)
N1 –C1	1.383(3)	N2–N3–C2	111.6(2)
N1–N2	1.316(3)	N1–N2–N3	104.6(2)
N2–N3	1.336(3)	N2–N1–C1	114.2(2)
C2–N3	1.376(3)	N1–C1–C2	103.1(2)
B1–N1	1.606(3)	N1–B1–C29	111.6(2)
B1–C29	1.633(3)	N1–B1–C15	102.5(2)
B1–C15	1.638(2)	C29–B1–C15	111.3(2)
B1–C35	1.655(3)	N1–B1–C35	102.6(2)
		C29–B1–C35	117.5(2)
		C15–B1–C35	110.0(2)

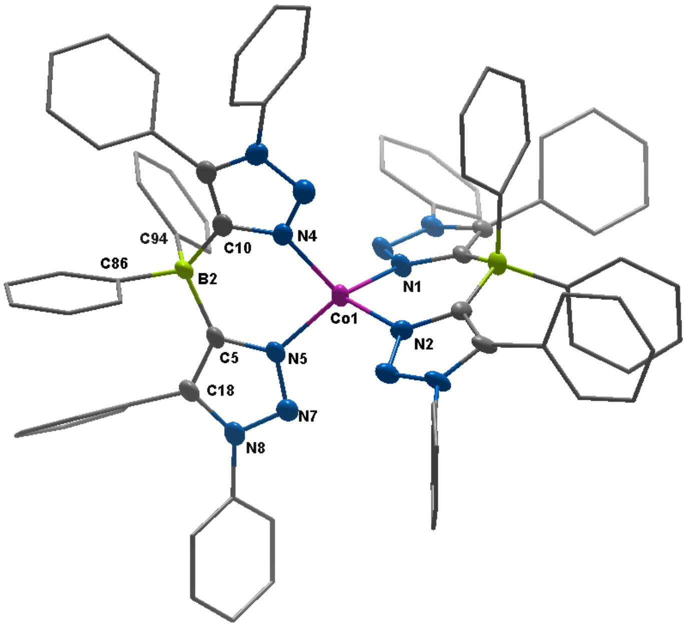

Figure S27: *ORTEP representation of [Co(**L2**$_{N,N}$)$_2$]: ellipsoids drawn at 50% probability. Solvent molecules and H-atoms omitted for clarity.*

Table S7: *Selected bond length and bond angles of [Co(**L2**$_{N,N}$)$_2$].*

Atom	Bond length (Å)	Atom	Bond angle (°)
Co1–N1	1.977(2)	N1–Co1–N5	119.4(1)
Co1–N2	1.965(3)	N2–Co1–N1	94.1(1)
Co1–N4	1.973(3)	N2–Co1–N4	118.1(1)
Co1–N5	1.976(3)	N2–Co1–N5	115.9(1)
N5–N7	1.321(4)	N4–Co1–N1	116.7(1)
N7–N8	1.334(4)	N4–Co1–N5	94.7(1)
N8–C18	1.364(5)	N5–N7–N8	105.3(3)
C5–C18	1.383(5)	N7–N8–C18	111.4(3)
N5–C5	1.372(4)	N8–C18–C5	106.3(3)
C5–B2	1.638(6)	N5–C5–C18	104.6(3)
C10–B2	1.655(6)	N7–N5–C5	112.5(3)
B2–C94	1.447(9)	C5–B2–C86	105.9(4)
B2–C86	1.77(1)	C10–B2–C86	98.4(4)
		C5–B2–C10	112.4(3)
		C94–B2–C10	106.1(4)
		C94–B2–C5	116.1(5)
		C94–B2–C86	116.7(5)

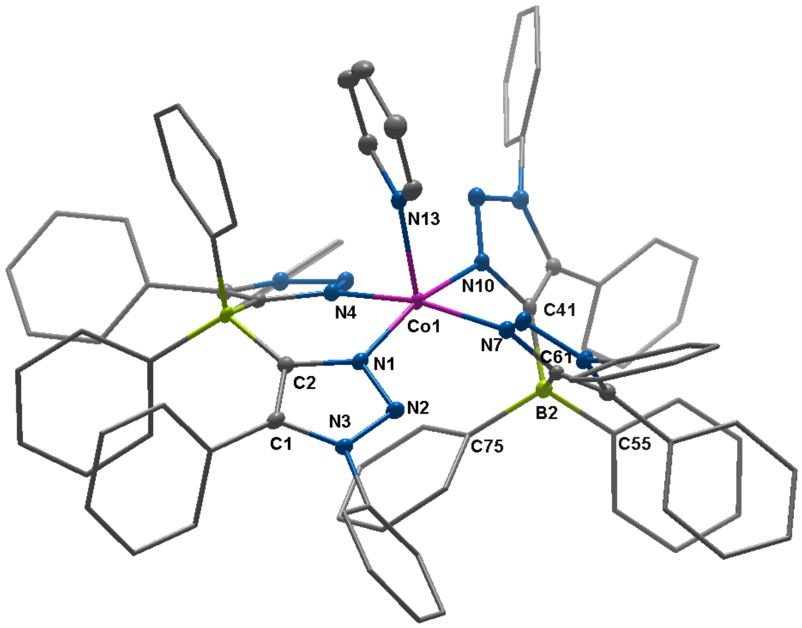

Figure S28: *ORTEP representation of [Co(**L2**$_{N,N}$)$_2$py]: ellipsoids drawn at 50% probability. Solvent molecules and H-atoms omitted for clarity.*

Table S8: *Selected bond length and bond angles of [Co(**L2**$_{N,N}$)$_2$py].*

Atom	Bond length (Å)	Atom	Bond angle (°)
Co1–N1	1.956(1)	N1–Co1–N4	89.13(5)
Co1–N4	1.969(1)	N1–Co1–N10	166.72(6)
Co1–N7	1.938(1)	N7–Co1–N1	91.21(5)
Co1–N10	1.956(1)	N7–Co1–N4	165.70(6)
Co1–N13	2.138(1)	N7–Co1–N10	84.48(5)
N1–N2	1.326(2)	N10–Co1–N4	91.97(5)
N2–N3	1.340(2)	N1–Co1–N13	96.50(5)
N3–C1	1.377(2)	N1–N2–N3	105.8(1)
C2–C1	1.401(2)	N2–N3–C1	111.7(1)
C41–B2	1.654(2)	N3–C1–C2	105.4(1)
C61–B2	1.652(2)	N1–C2–C1	105.0(1)
C55–B2	1.634(2)	N2–N1–C2	112.0(1)
C75–B2	1.649(2)	C61–B2–C41	103.7(1)
		C55–B2–C41	116.0(1)
		C75–B2–C41	106.9(1)
		C55–B2–C61	109.5(1)
		C55–B2–C75	111.2(1)
		C75–B2–C61	109.0(1)

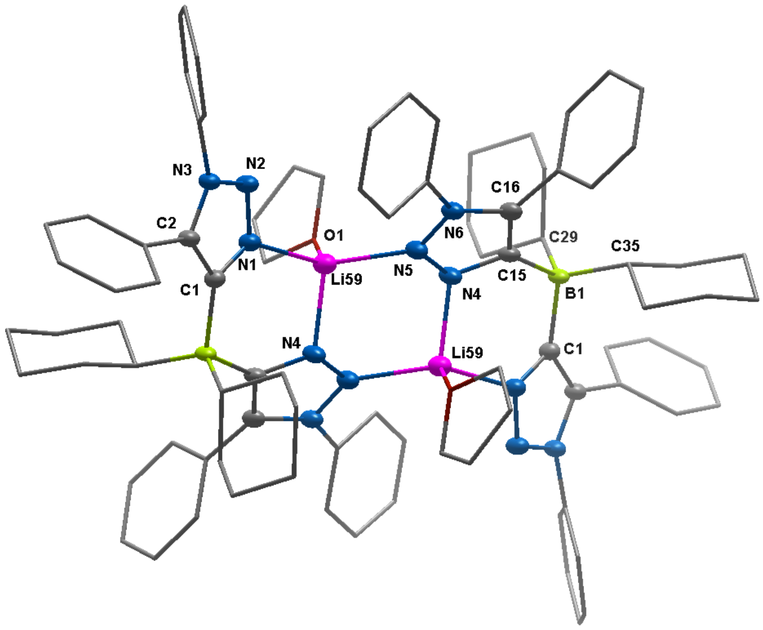

Figure S29: *ORTEP representation of [Li(**L1**$_{N,N}$)]$_2$: ellipsoids drawn at 50% probability. Solvent molecules and H-atoms omitted for clarity.*

Table S9: *Selected bond length and bond angles of [Li(**L1**$_{N,N}$)]$_2$.*

Atom	Bond length (Å)	Atom	Bond angle (°)
N1–Li59	1.976(4)	N1–Li59–N5	137.3(2)
N5–Li59	2.039(4)	N4–Li59–N5	103.8(2)
N4–Li59	2.026(4)	N1–Li59–N4	91.9(2)
O1–Li59	1.943(4)	N1–N2–N3	105.9(2)
N2–N1	1.314(2)	N2–N3–C2	111.0(2)
N3–N2	1.344(2)	N3–C2–C1	106.0(2)
N3–C2	1.367(3)	N1–C1–C2	104.7(2)
C2–C1	1.395(3)	N2–N1–C1	112.4(2)
N1–C1	1.375(3)	C15–B1–C29	107.3(2)
B1–C1	1.664(3)	C15–B1–C35	108.1(2)
B1–C15	1.652(3)	C15–B1–C1	109.0(2)
C29–B1	1.658(3)	C35–B1–C29	114.2(2)
C35–B1	1.655(3)	C35–B1–C1	109.2(2)
		C29–B1–C1	108.9(2)

+ **Table S10:** *Crystallographic details.*

	[Co($\mathbf{L1}_{N,N}$)$_2$]	[Co($\mathbf{L1}_{C,N}$)$_2$]	[Co($\mathbf{L2}_{N,N}$)$_2$]
Chemical formula	$C_{80}H_{84}B_2CoN_{12}$	$C_{80}H_{84}B_2CoN_{12}$	$C_{80}H_{60}B_2CoN_{12}$
M_r	725.63	395.64	1269.95
Crystal system	Triclinic	Triclinic	Monoclinic
Space group	$P\bar{1}$	$P\bar{1}$	$P21/c$
a (Å)	12.3891(9)	11.600(2)	23.0013(7)
b (Å)	12.702(1)	14.319(3)	20.3174(6)
c (Å)	13.493(1)	15.169(3)	19.5333(7)
α (°)	95.865(3)	61.927(4)	90
β (°)	90.227(3)	70.819(7)	112.689(1)
γ (°)	118.977(2)	72.828(5)	90
V (Å^3)	1844.3(3)	2069.6(7)	8422.0(5)
Z	2	4	4
Densitiy (g cm^{-3})	1.307	1.270	1.002
F(000)	769	845	2644
Radiation Type	MoK$_\alpha$	MoK$_\alpha$	MoK$_\alpha$
μ (mm^{-1})	0.350	0.269	0.248
Crystal size	0.29 x 0.21 x 0.11	0.15 x 0.11 x 0.09	0.43 x 0.24 x 0.09
Meas. Refl.	83869	89811	65956
Indep. Refl.	6801	8519	15428
Obsvd. [$I > 2\sigma(I)$] refl.	6162	7266	10605
R_{int}	0.0412	0.0562	0.1140
R [$F^2 > 2\sigma(F^2)$], wR(F^2), S	0.0368, 0.0894, 1.053	0.0551, 0.1473, 0.0664	0.0788, 0.1756, 1.033
$\Delta\rho_{max}$, $\Delta\rho_{min}$ (e Å^{-3})	0.631, –0.359	2.182, –0.958	0.858, –0.516
CCDC	2020849	2020850	2020851

	[Co(**L2**$_{N,N}$)$_2$py]	[Li(**L1**$_{C,C}$)]$_2$
Chemical formula	$C_{85}H_{65}B_2CoN_{13}$	$C_{88}H_{100}B_2Li_2N_{12}O_2$
M_r	1349.05	1577.56
Crystal system	Monoclinic	Triclinic
Space group	$P21/c$	$P\bar{1}$
a (Å)	12.7419(6)	12.2854(5)
b (Å)	22.708(1)	13.5549(6)
c (Å)	25.6716(8)	14.0523(5)
α (°)	90	97.913(3)
β (°)	95.441(2)	91.913(3)
γ (°)	90	105.115(4)
V (Å^3)	7394.3(5)	2235.8(2)
Z	4	1
Densitiy (g cm^{-3})	1.212	1.172
F(000)	2812	844
Radiation Type	MoK$_\alpha$	CuK$_\alpha$
μ (mm^{-1})	0.286	0.538
Crystal size	0.30 x 0.10 x 0.05	0.35 x 0.25 x 0.2
Meas. Refl.	137841	20812
Indep. Refl.	14989	8777
Obsvd. [$I > 2\sigma(I)$] refl.	13081	6913
R_{int}	0.0571	0.0957
R [$F^2 > 2\sigma(F^2)$], wR(F^2), S	0.0405, 0.0920, 0.0920	0.0869, 0.2564, 1.046
$\Delta\rho_{max}$, $\Delta\rho_{min}$ (e Å^{-3})	0.357, –0.514	0.630, –0.526
CCDC	2020520	2020853 (CSD)

8. Molecular Geometries used in Electronic Structure Calculations

[Co(L1$_{N,N}$)$_2$]

Co	3.09093	4.75329	6.69841
N	2.75548	6.53525	7.28894
N	3.42638	2.97132	6.10787
N	3.64759	7.47894	7.06709
N	2.53427	2.02764	6.32973
N	2.40586	4.18213	8.39685
N	3.77600	5.32445	4.99996
N	3.00626	3.19035	9.02369
N	3.17560	6.31623	4.37312
C	1.66030	6.97605	7.99937
C	4.52156	2.53053	5.39744
N	3.14010	8.57228	7.63069
N	3.04176	0.93429	5.76612
C	1.24235	4.60288	9.01954
C	4.93950	4.90370	4.37727
N	2.21808	2.91715	10.06261
N	3.96378	6.58943	3.33420
B	0.44341	5.93987	8.43946
C	1.92046	8.32739	8.21184
B	5.73845	3.56671	4.95736
C	4.26139	1.17919	5.18497
C	4.10051	9.72270	7.85723
C	2.40448	-0.32122	6.01785
C	1.11405	3.73279	10.09691
C	5.06781	5.77379	3.29990
C	2.63612	1.90397	10.99503
C	3.54573	7.60261	2.40178
C	-0.43704	5.50500	7.10379
C	-0.51098	6.72604	9.52996
C	1.32259	9.44487	9.04419
C	6.61889	4.00157	6.29302
C	6.69284	2.78054	3.86686
C	5.11375	0.06960	4.74113
C	3.63303	10.96028	7.50757
C	5.38287	9.48875	8.28861
C	1.34214	-0.61076	5.18859
C	2.77312	-1.18046	7.03065
C	0.09502	3.49004	11.14508
C	6.08684	6.01654	2.25174
C	3.55477	2.21543	11.94554
C	2.13664	0.64269	10.91840
C	2.62708	7.29115	1.45128
C	4.04522	8.86388	2.47841
C	0.26422	4.60932	6.08336
C	-1.02580	6.71996	6.36349
C	-1.92729	6.16704	9.76869
C	0.15315	7.01186	10.88531
C	1.93162	9.92016	10.20569
C	0.17307	10.05635	8.57798
C	5.91764	4.89726	7.31345
C	7.20766	2.78662	7.03333
C	8.10915	3.33954	3.62813
C	6.02871	2.49472	2.51150
C	4.58810	-0.59024	3.63589
C	6.31076	-0.36165	5.27968
C	6.26247	10.55465	8.35157
C	4.52437	12.03251	7.58260

C	0.67677	-1.80003	5.37614
C	2.07992	-2.36955	7.20749
C	0.38699	3.75502	12.47779
C	-1.13877	2.95008	10.81980
C	5.79487	5.75156	0.91902
C	7.32063	6.55650	2.57701
C	3.98655	1.23598	12.83107
C	2.58398	-0.32354	11.81331
C	2.19531	8.27060	0.56575
C	3.59788	9.83012	1.58350
C	-0.64278	4.20946	4.91824
C	-1.91143	6.34796	5.17546
C	-2.76648	7.07388	10.67565
C	-0.68510	7.93851	11.77446
C	1.39238	10.99586	10.88223
C	-0.36539	11.16176	9.23844
C	6.82464	5.29712	8.47857
C	8.09329	3.15861	8.22136
C	8.94834	2.43269	2.72116
C	6.86696	1.56807	1.62235
C	5.27669	-1.69230	3.08663
C	6.98889	-1.42517	4.75185
C	5.82352	11.81127	8.00995
C	1.02062	-2.67940	6.37152
C	-0.55024	3.50552	13.47291
C	-2.06819	2.69535	11.82604
C	6.73210	6.00106	-0.07609
C	8.25005	6.81122	1.57078
C	3.49927	-0.02901	12.75109
C	2.68259	9.53558	0.64573
C	-1.18079	5.43801	4.19990
C	-2.06703	7.33626	12.00408
C	0.25412	11.62293	10.39727
C	7.36264	4.06857	9.19691
C	8.24889	2.17031	1.39273
C	6.43844	-2.08359	3.65331
C	-1.76714	2.97491	13.14562
C	7.94900	6.53167	0.25119
H	-1.21233	4.91133	7.54127
H	-0.64099	7.64675	9.00054
H	7.39418	4.59523	5.85553
H	6.82285	1.85983	4.39628
H	2.65745	11.09621	7.19969
H	5.68365	8.53950	8.55967
H	1.05383	0.04675	4.44726
H	3.56007	-0.94087	7.65381
H	3.93010	3.17462	12.00966
H	1.43213	0.40392	10.20308
H	2.25174	6.33197	1.38717
H	4.74973	9.10264	3.19373
H	1.11027	5.13491	5.69240
H	0.55164	3.71116	6.58894
H	-1.61184	7.28612	7.05697
H	-0.20003	7.27797	5.97411
H	-1.84408	5.20397	10.22746
H	-2.41938	6.10757	8.82042
H	1.10264	7.47306	10.71022
H	0.25382	6.07805	11.39792
H	2.78791	9.46623	10.56028
H	-0.29187	9.69115	7.73212
H	5.07158	4.37168	7.70441
H	5.63023	5.79542	6.80787

H	7.79370	2.22046	6.33985
H	6.38188	2.22862	7.42272
H	8.02594	4.30261	3.16936
H	8.60124	3.39902	4.57640
H	5.07922	2.03352	2.68659
H	5.92804	3.42853	1.99889
H	3.69810	-0.27520	3.21914
H	6.70174	0.12987	6.09856
H	7.23871	10.40755	8.65214
H	4.21767	12.98270	7.32166
H	-0.10639	-2.03850	4.74779
H	2.35316	-3.02094	7.95986
H	1.30985	4.14127	12.73109
H	-1.36837	2.73747	9.83639
H	4.87201	5.36532	0.66572
H	7.55023	6.76912	3.56042
H	4.68300	1.47155	13.55528
H	2.20650	-1.28230	11.75581
H	1.49886	8.03503	-0.15846
H	3.97536	10.78888	1.64099
H	-0.08227	3.61671	4.22587
H	-1.46959	3.65159	5.30563
H	-2.78307	5.84288	5.53608
H	-2.17837	7.24722	4.66071
H	-3.70736	6.60101	10.86551
H	-2.90479	8.01103	10.17813
H	-0.78883	8.88972	11.29557
H	-0.19389	8.05706	12.71762
H	1.83678	11.33400	11.75010
H	-1.20956	11.63010	8.87367
H	6.26413	5.88988	9.17094
H	7.65145	5.85498	8.09118
H	8.96493	3.66368	7.86075
H	8.36022	2.25935	8.73611
H	9.88922	2.90556	2.53130
H	9.08664	1.49554	3.21868
H	6.97068	0.61686	2.10124
H	6.37575	1.44952	0.67919
H	4.89907	-2.19096	2.26580
H	7.88670	-1.73307	5.15701
H	6.47631	12.60808	8.07316
H	0.49679	-3.55989	6.49546
H	-0.32814	3.72040	14.45755
H	-2.98812	2.29500	11.58425
H	6.51000	5.78618	-1.06073
H	9.16999	7.21156	1.81258
H	3.82893	-0.75677	13.40433
H	2.35293	10.26333	-0.00751
H	-1.85860	5.12833	3.43206
H	-0.36051	5.97609	3.77270
H	-1.96429	6.41326	12.53551
H	-2.65408	8.02753	12.57190
H	-0.13611	12.43634	10.89837
H	8.04044	4.37825	9.96475
H	6.54235	3.53049	9.62410
H	8.14615	3.09331	0.86130
H	8.83594	1.47904	0.82491
H	6.93563	-2.89776	3.25968
H	-2.46146	2.78300	13.88462
H	8.64331	6.72359	-0.48781

[Co(L1$_{N,N}$)$_2$]^{Tr}

Co	3.09093	4.75329	6.69841
N	2.75548	6.53525	7.28894
N	3.42638	2.97132	6.10787
N	3.64759	7.47894	7.06709
N	2.53427	2.02764	6.32973
N	2.40586	4.18213	8.39685
N	3.77600	5.32445	4.99996
N	3.00626	3.19035	9.02369
N	3.17560	6.31623	4.37312
C	1.66030	6.97605	7.99937
C	4.52156	2.53053	5.39744
N	3.14010	8.57228	7.63069
N	3.04176	0.93429	5.76612
C	1.24235	4.60288	9.01954
C	4.93950	4.90370	4.37727
N	2.21808	2.91715	10.06261
N	3.96378	6.58943	3.33420
B	0.44341	5.93987	8.43946
C	1.92046	8.32739	8.21184
B	5.73845	3.56671	4.95736
C	4.26139	1.17919	5.18497
C	4.10051	9.72270	7.85723
C	2.40448	-0.32122	6.01785
C	1.11405	3.73279	10.09691
C	5.06781	5.77379	3.29990
C	2.63612	1.90397	10.99503
C	3.54573	7.60261	2.40178
C	-0.43704	5.50500	7.10379
C	-0.51098	6.72604	9.52996
C	1.32259	9.44487	9.04419
C	6.61889	4.00157	6.29302
C	6.69284	2.78054	3.86686
C	5.11375	0.06960	4.74113
C	3.63303	10.96028	7.50757
C	5.38287	9.48875	8.28861
C	1.34214	-0.61076	5.18859
C	2.77312	-1.18046	7.03065
C	0.09502	3.49004	11.14508
C	6.08684	6.01654	2.25174
C	3.55477	2.21543	11.94554
C	2.13664	0.64269	10.91840
C	2.62708	7.29115	1.45128
C	4.04522	8.86388	2.47841
C	-1.02580	6.71996	6.36349
C	-1.92729	6.16704	9.76869
C	0.15315	7.01186	10.88531
C	1.93162	9.92016	10.20569
C	0.17307	10.05635	8.57798
C	7.20766	2.78662	7.03333
C	8.10915	3.33954	3.62813
C	6.02871	2.49472	2.51150
C	4.58810	-0.59024	3.63589
C	6.31076	-0.36165	5.27968
C	6.26247	10.55465	8.35157
C	4.52437	12.03251	7.58260
C	0.67677	-1.80003	5.37614
C	2.07992	-2.36955	7.20749
C	0.38699	3.75502	12.47779
C	-1.13877	2.95008	10.81980
C	5.79487	5.75156	0.91902

C	7.32063	6.55650	2.57701
C	3.98655	1.23598	12.83107
C	2.58398	-0.32354	11.81331
C	2.19531	8.27060	0.56575
C	3.59788	9.83012	1.58350
C	-0.64278	4.20946	4.91824
C	-1.91143	6.34796	5.17546
C	-2.76648	7.07388	10.67565
C	-0.68510	7.93851	11.77446
C	1.39238	10.99586	10.88223
C	-0.36539	11.16176	9.23844
C	6.82464	5.29712	8.47857
C	8.09329	3.15861	8.22136
C	8.94834	2.43269	2.72116
C	6.86696	1.56807	1.62235
C	5.27669	-1.69230	3.08663
C	6.98889	-1.42517	4.75185
C	5.82352	11.81127	8.00995
C	1.02062	-2.67940	6.37152
C	-0.55024	3.50552	13.47291
C	-2.06819	2.69535	11.82604
C	6.73210	6.00106	-0.07609
C	8.25005	6.81122	1.57078
C	3.49927	-0.02901	12.75109
C	2.68259	9.53558	0.64573
C	-1.18079	5.43801	4.19990
C	-2.06703	7.33626	12.00408
C	0.25412	11.62293	10.39727
C	7.36264	4.06857	9.19691
C	8.24889	2.17031	1.39273
C	6.43844	-2.08359	3.65331
C	-1.76714	2.97491	13.14562
C	7.94900	6.53167	0.25119
H	-1.21335	4.85147	7.46005
H	-0.64099	7.64675	9.00054
H	7.31751	4.71846	5.91267
H	6.82285	1.85983	4.39628
H	2.65745	11.09621	7.19969
H	5.68365	8.53950	8.55967
H	1.05383	0.04675	4.44726
H	3.56007	-0.94087	7.65381
H	3.93010	3.17462	12.00966
H	1.43213	0.40392	10.20308
H	2.25174	6.33197	1.38717
H	4.74973	9.10264	3.19373
H	-1.61184	7.28612	7.05697
H	-0.20003	7.27797	5.97411
H	-1.84408	5.20397	10.22746
H	-2.41938	6.10757	8.82042
H	1.10264	7.47306	10.71022
H	0.25382	6.07805	11.39792
H	2.78791	9.46623	10.56028
H	-0.29187	9.69115	7.73212
H	7.79370	2.22046	6.33985
H	6.38188	2.22862	7.42272
H	8.02594	4.30261	3.16936
H	8.60124	3.39902	4.57640
H	5.07922	2.03352	2.68659
H	5.92804	3.42853	1.99889
H	3.69810	-0.27520	3.21914
H	6.70174	0.12987	6.09856
H	7.23871	10.40755	8.65214

H	4.21767	12.98270	7.32166
H	-0.10639	-2.03850	4.74779
H	2.35316	-3.02094	7.95986
H	1.30985	4.14127	12.73109
H	-1.36837	2.73747	9.83639
H	4.87201	5.36532	0.66572
H	7.55023	6.76912	3.56042
H	4.68300	1.47155	13.55528
H	2.20650	-1.28230	11.75581
H	1.49886	8.03503	-0.15846
H	3.97536	10.78888	1.64099
H	-0.08227	3.61671	4.22587
H	-1.46959	3.65159	5.30563
H	-2.78307	5.84288	5.53608
H	-2.17837	7.24722	4.66071
H	-3.70736	6.60101	10.86551
H	-2.90479	8.01103	10.17813
H	-0.78883	8.88972	11.29557
H	-0.19389	8.05706	12.71762
H	1.83678	11.33400	11.75010
H	-1.20956	11.63010	8.87367
H	6.26413	5.88988	9.17094
H	7.65145	5.85498	8.09118
H	8.96493	3.66368	7.86075
H	8.36022	2.25935	8.73611
H	9.88922	2.90556	2.53130
H	9.08664	1.49554	3.21868
H	6.97068	0.61686	2.10124
H	6.37575	1.44952	0.67919
H	4.89907	-2.19096	2.26580
H	7.88670	-1.73307	5.15701
H	6.47631	12.60808	8.07316
H	0.49679	-3.55989	6.49546
H	-0.32814	3.72040	14.45755
H	-2.98812	2.29500	11.58425
H	6.51000	5.78618	-1.06073
H	9.16999	7.21156	1.81258
H	3.82893	-0.75677	13.40433
H	2.35293	10.26333	-0.00751
H	-1.85860	5.12833	3.43206
H	-0.36051	5.97609	3.77270
H	-1.96429	6.41326	12.53551
H	-2.65408	8.02753	12.57190
H	-0.13611	12.43634	10.89837
H	8.04044	4.37825	9.96475
H	6.54235	3.53049	9.62410
H	8.14615	3.09331	0.86130
H	8.83594	1.47904	0.82491
H	6.93563	-2.89776	3.25968
H	-2.46146	2.78300	13.88462
H	8.64331	6.72359	-0.48781
H	5.77868	4.17281	6.90341
H	6.09101	5.04172	7.74887
H	0.16640	4.42806	5.58656
H	0.30234	5.13032	6.49910

[Co(**L2**$_{N,N}$)$_2$py]

Co	7.50349	11.45728	7.32688
N	7.07259	12.26705	5.59963
N	9.03815	10.55178	6.48868
N	5.76392	11.94336	8.03208

N	7.62953	10.33980	8.92675
N	8.65033	13.09538	8.08095
N	5.80366	12.55829	5.35700
C	7.88505	12.52915	4.50905
N	9.41997	9.41113	7.04206
C	9.76692	10.83954	5.34034
N	5.26389	13.15905	8.11682
C	4.84239	10.95589	8.32977
N	8.67457	10.26687	9.72652
C	6.69733	9.33894	9.16285
C	9.93447	12.89881	8.44449
C	8.18695	14.35922	8.12865
N	5.76778	13.02493	4.10305
C	9.89771	11.89565	2.88589
C	7.02374	13.04786	3.53280
C	10.37337	13.44988	5.04468
C	7.24171	13.55224	2.15545
B	9.49117	12.19643	4.44940
N	10.41400	8.94087	6.27952
C	10.68053	9.79056	5.22092
C	11.86301	9.52062	4.35264
N	3.97229	12.97947	8.45958
C	3.66775	11.64625	8.60779
C	5.73125	8.99457	6.72080
C	2.26966	11.22665	8.87842
C	4.09390	8.42361	8.80534
B	5.31181	9.37165	8.27256
N	8.45402	9.18606	10.50346
C	7.26028	8.56903	10.18145
C	6.84877	7.29317	10.80984
C	10.78479	13.92294	8.83987
C	10.29448	15.22084	8.88936
C	8.96390	15.43625	8.53170
C	4.46588	13.36617	3.56473
C	9.11434	11.02782	2.09571
C	11.01863	12.45903	2.26154
C	9.43404	10.74788	0.76142
C	11.35663	12.17364	0.93570
C	6.52523	13.01396	1.07782
C	8.16334	14.57148	1.92345
C	9.83210	14.72958	5.24111
C	11.75516	13.31850	5.30627
C	10.61270	15.81819	5.64595
C	12.55077	14.39803	5.70064
C	6.73592	13.49901	-0.20982
C	8.35994	15.06403	0.63656
C	10.90694	7.62247	6.58986
C	13.12938	9.40417	4.94261
C	11.73603	9.27106	2.99914
C	14.24925	9.04859	4.18317
C	12.87470	8.93979	2.24277
C	3.14106	14.14879	8.61021
C	1.30864	11.38635	7.87910
C	1.89560	10.70116	10.11545
C	6.75160	8.06825	6.43071
C	5.06055	9.53013	5.60299
C	5.38558	9.18546	4.29297
C	7.09110	7.71224	5.12938
C	-0.01566	11.00309	8.11091
C	0.57094	10.31655	10.33755
C	3.84315	8.17629	10.16535
C	2.72202	7.46058	10.60515

C	3.14487	7.89299	7.90811
C	2.00683	7.20340	8.32466
C	9.47945	8.80158	11.44220
C	6.48108	6.23262	9.97674
C	6.84376	7.10922	12.19852
C	6.14220	4.99243	10.51163
C	6.48342	5.87153	12.73136
C	3.42534	12.45478	3.76096
C	4.25857	14.56727	2.90505
C	2.14901	12.76582	3.27529
C	2.98057	14.86310	2.42347
C	10.56198	11.32442	0.18360
C	7.64621	14.52461	-0.42981
C	11.97962	15.66048	5.86307
C	11.34602	6.77138	5.58082
C	10.86815	7.19828	7.91869
C	11.77482	5.48681	5.92233
C	11.25791	5.89827	8.22877
C	14.11700	8.83083	2.83121
C	2.38595	14.32858	9.76705
C	3.12525	15.09451	7.58608
C	2.36294	16.24771	7.73544
C	1.61165	15.48814	9.89520
C	-0.38244	10.46649	9.33847
C	6.41520	8.27414	4.05272
C	1.79804	6.98628	9.68570
C	10.00457	7.51435	11.38837
C	9.94915	9.74428	12.35476
C	10.98794	9.39001	13.22015
C	11.04504	7.17302	12.26296
C	6.14292	4.81484	11.89366
C	1.93249	13.96305	2.60514
C	11.72236	5.04219	7.23430
C	1.60347	16.44453	8.88462
C	11.53843	8.10119	13.16761
H	10.28805	11.85839	8.41103
H	7.13218	14.49183	7.84235
H	11.82566	13.68974	9.10726
H	10.93881	16.05877	9.19621
H	8.52285	16.44396	8.55160
H	8.22381	10.55178	2.54148
H	11.66094	13.15023	2.82984
H	8.79380	10.07153	0.17101
H	12.25192	12.63485	0.48581
H	5.81272	12.19243	1.24680
H	8.73289	14.98819	2.76630
H	8.75184	14.88865	5.08705
H	12.23064	12.32942	5.18917
H	10.14264	16.80460	5.79343
H	13.62814	14.25018	5.88383
H	6.17831	13.06410	-1.05420
H	9.08654	15.87341	0.46519
H	13.24318	9.58789	6.02259
H	10.75634	9.32940	2.51087
H	15.23037	8.96117	4.67550
H	12.75670	8.75727	1.16295
H	1.59837	11.80457	6.90243
H	2.64548	10.59011	10.91035
H	7.32772	7.62806	7.25983
H	4.25595	10.26179	5.78184
H	4.83875	9.63219	3.44606
H	7.90599	6.99068	4.95214

H	-0.76496	11.12707	7.31323
H	0.28699	9.89244	11.31323
H	4.53360	8.57598	10.92540
H	2.56806	7.29211	11.68384
H	3.29439	8.04865	6.82661
H	1.28241	6.82860	7.58309
H	6.47942	6.37968	8.88736
H	7.11028	7.94071	12.86817
H	5.86864	4.16189	9.84288
H	6.47374	5.73345	13.82393
H	3.61677	11.51473	4.29200
H	5.08415	15.28081	2.77551
H	1.32493	12.05145	3.42419
H	2.80800	15.81689	1.90209
H	10.81849	11.10560	-0.86635
H	7.80617	14.90884	-1.44982
H	12.59836	16.51779	6.17459
H	11.34549	7.08819	4.53129
H	10.52170	7.89353	8.69330
H	12.12995	4.81579	5.12494
H	11.22130	5.55666	9.27487
H	14.99481	8.56121	2.22254
H	2.41270	13.57978	10.57061
H	3.72252	14.91723	6.67992
H	2.35543	16.99935	6.93116
H	1.01597	15.63958	10.80832
H	-1.42512	10.16094	9.51891
H	6.68680	8.00224	3.01971
H	0.90544	6.43722	10.02917
H	9.60941	6.78114	10.67266
H	9.50855	10.75109	12.37506
H	11.37046	10.12756	13.94184
H	11.47058	6.15842	12.22174
H	5.87129	3.83821	12.32453
H	0.92980	14.20539	2.22036
H	12.04535	4.02126	7.48850
H	0.99722	17.35668	8.99529
H	12.35833	7.82735	13.84896

9. References

[1] G. R. Fulmer, A. J. M. Miller, N. H. Sherden, H. E. Gottlieb, A. Nudelman, B. M. Stoltz, J. E. Bercaw, K. I. Goldberg, *Organometallics* **2010**, *29*, 2176–2179.

[2] A. L. Spek, *J. Appl. Crystallogr.* **2003**, *36*, 7–13.

[3] G. M. Sheldrick, *Acta Crystallogr., Sect. C: Struct. Chem.* **2015**, *71*, 3–8.

[4] G. M. Sheldrick, *Acta Crystallogr., Sect. A: Found. Crystallogr.* **2008**, *64*, 112–122.

[5] G. M. Sheldrick, *SHELXS-97, Program for Crystal Structure Solution and Refinement*, University of Göttingen, Göttingen, Germany, **1997**.

[6] G. M. Sheldrick, *SHELXL Version 2014/7, Program for Chrystal Structure Solution and Refinement*, University of Göttingen, Germany, **2014**.

[7] G. M. Sheldrick, *SADABS Ver. 2008/1*, *SADABS. Program for Empirical Absorption Correction*, University of Gottingen, Germany, **2012**.

[8] SAINT+, *Data Integration Engine, Version 8.27b©*, Bruker AXS Inc., Madison, Wisconsin, USA, **1997-2012**.

[9] O. V. Dolomanov, L. J. Bourhis, R. J. Gildea, J. A. K. Howard, H. Puschmann, *J. Appl. Crystallogr.* **2009**, *42*, 339–341.

[10] S. W. Kwok, J. R. Fotsing, R. J. Fraser, V. O. Rodionov, V. V. Fokin, *Org. Lett.* **2010**, *12*, 4217-4219.

[11] J. C. Thomas, J. C. Peters, *Inorg. Chem.* **2003**, *42*, 5055-5073.

[12] A. K. Hickey, W.-T. Lee, C.-H. Chen, M. Pink, J. M. Smith, *Organometallics* **2016**, *35*, 3069–3073.

[13] N. I. Neuman, M. Perec, P. J. González, M. C. G. Passeggi, A. C. Rizzi, C. D. Brondino, *J. Phys. Chem. A* **2010**, *114*, 13069–13075.

[14] N. I. Neuman, V. G. Franco, F. M. Ferroni, R. Baggio, M. C. Passeggi, A. C. Rizzi, C. D. Brondino, *J. Phys. Chem. A* **2012**, *116*, 12314–12320.

[15] A. C. Rizzi, N. I. Neuman, P. J. González, C. D. Brondino, *Eur. J. Inorg. Chem.* **2016**, *2016*, 192–207.

[16] N. I. Neuman, E. Winkler, O. Pena, M. C. Passeggi, A. C. Rizzi, C. D. Brondino, *Inorg. Chem.* **2014**, *53*, 2535–2544.

[17] N. I. Neuman, E. Burna, R. Baggio, M. C. G. Passeggi, A. C. Rizzi, C. D. Brondino, *Inorg. Chem. Front.* **2015**, *2*, 837–845.

[18] F. Neese, *WIREs Comput Mol Sci* **2018**, *8*, 1327–1332.

[19] B. O. Roos, P. R. Taylor, *Chem. Phys.* **1980**, *48*, 157–173.

[20] P. E. M. Siegbahn, J. Almlöf, A. Heiberg, B. O. Roos, *J. Chem. Phys.* **1981**, *74*, 2384–2396.

[21] C. Angeli, R. Cimiraglia, S. Evangelisti, T. Leininger, J.-P. Malrieu, *J. Chem. Phy.* **2001**, *114*, 10252–10264.

[22] C. Angeli, R. Cimiraglia, J.-P. Malrieu, *Chem. Phys. Lett.* **2001**, *350*, 297–305.

[23] C. Angeli, R. Cimiraglia, J.-P. Malrieu, *J. Chem. Phy.* **2002**, *117*, 9138–9153.

[24] F. Weigend, R. Ahlrichs, *Phys. Chem. Chem. Phys.* **2005**, *7*, 3297–3305.

[25] T. Petrenko, S. Kossmann, F. Neese, *J. Chem. Phy.* **2011**, *134*, 054116.

[26] F. Neese, G. Olbrich, *Chem. Phys. Lett.* **2002**, *362*, 170–178.

[27] R. Izsak, F. Neese, *J. Chem. Phy.* **2011**, *135*, 144105.

[28] J. L. Whitten, *J. Chem. Phy.* **1973**, *58*, 4496–4501.

[29] O. Vahtras, J. Almlöf, M. W. Feyereisen, *Chem. Phys. Lett.* **1993**, *213*, 514–518.

[30] F. Neese, F. Wennmohs, A. Hansen, U. Becker, *Chem. Phys.* **2009**, *356*, 98–109.

[31] F. Neese, *J. Comput. Chem.* **2003**, *24*, 1740–1747.

[32] K. Eichkorn, O. Treutler, H. Öhm, M. Häser, R. Ahlrichs, *Chem. Phys. Lett.* **1995**, *242*, 652–660.

[33] K. Eichkorn, F. Weigend, O. Treutler, R. Ahlrichs, *Theor. Chem. Acc.* **1997**, *97*, 119–124.

[34] F. Weigend, *Phys. Chem. Chem. Phys.* **2006**, *8*, 1057–1065.

[35] J. P. Perdew, K. Burke, M. Ernzerhof, *Phys. Rev. Lett.* **1996**, *77*, 3865–3868.

[36] Chemcraft – graphical software for visualization of quantum chemistry computations, https://www.chemcraftprog.com.).

[37] A. H. Maki, N. Edelstein, A. Davison, R. H. Holm, *J. Am. Chem. Soc.* **1964**, *86*, 4580-4587.

[38] B. R. McGarvey, *Can. J. Chem.* **1975**, *53*, 2498-2511.
